# Trends of cervical cancer at global, regional, and national level: data from the Global Burden of Disease study 2019

**DOI:** 10.1186/s12889-021-10907-5

**Published:** 2021-05-12

**Authors:** Xingxing Zhang, Qingle Zeng, Wenwen Cai, Weiqing Ruan

**Affiliations:** 1 Huiqiao Medical Center, Nanfang Hospital, Southern Medical University, Guangzhou, Guangdong China; 2 Department of Interventional Radiology, Nanfang Hospital, Southern Medical University, Guangzhou, China

**Keywords:** Cervical cancer, Global burden of disease, Quality-adjusted life years, Global Health, Health services

## Abstract

**Background:**

Cervical cancer is an important global health problem. In this study we aimed to analyze trends in cervical cancer at the global, regional, and national levels from 1990 to 2019, to inform health service decision-making.

**Methods:**

Data on cervical cancer was extracted from the Global Burden of Disease study, 2019. Trends in cervical cancer burden were assessed based on estimated annual percentage change (EAPC) and age-standardized rate (ASR).

**Results:**

Globally, decreasing trends were observed in incidence, death, and disability adjusted life years (DALYs) of cervical cancer from 1990 to 2019, with respective EAPCs of − 0.38 (95% confidence interval [CI]: − 0.41 to − 0.34), − 0.93 (95%CI: − 0.98 to − 0.88), and − 0.95 (95 CI%: − 1.00 to − 0.90). Meanwhile, decreasing trends were detected in most sociodemographic index (SDI) areas and geographic regions, particularly death and DALYs in Central Latin America, with respective EAPCs of − 2.61 (95% CI: − 2.76 to − 2.46) and − 2.48 (95% CI: − 2.63 to − 2.32); hhowever, a pronounced increasing trend in incidence occurred in East Asia (EAPC = 1.33; 95% CI: 1.12 to 1.55). At the national level, decreasing trends in cervical cancer were observed in most countries/territories, particularly DALYs in the Maldives (EAPC = − 5.06; 95% CI: − 5.40 to − 4.72), Whereas increasing trends were detected in Lesotho, Zimbabwe, and Bulgaria.

**Conclusions:**

Slowly decreasing trends in cervical cancer were detected worldwide from 1990 to 2019. Cervical cancer remains a substantial health problem for women globally, requiring more effective prevention and control strategies.

**Supplementary Information:**

The online version contains supplementary material available at 10.1186/s12889-021-10907-5.

## Introduction

Cervical cancer is the fourth leading cause of cancer death among women worldwide [1, 2]. It is estimated that there were 570,000 new cases and 311,000 deaths of women (particularly middle-aged women) from cervical cancer globally in 2018 [3, 4].

The distribution of cervical cancer differs across the world, with than 85% of deaths occurring in developing regions [5]. Over 90% of the highest incidence rates of cervical cancer occur in sub-Saharan Africa [6]. The drastic changes in epidemiological patterns of cervical cancer, over recent decades have been attributed to the effectiveness of the Papanicolaou (Pap) test in improving detection of the human papilloma virus (HPV) [7]. For example, Finland launched an nationwide screening programmer for cervical cancer in 1960, which led to a steep decrease in the rate of cervical cancer (to below 1/100,000) from 1973 [8].

Australia has established an HPV vaccination program covering more than 70% of girls and boys aged 12–13 years nationwide, and the incidence of high-grade cervical dysplasia in girls < 18 years old reduced by 38% [9]. In contrast, the incidence of cervical cancer in the former Soviet Union has risen significantly, due to weak health care systems and inadequate screening programs [10]. Currently, three types of prophylactic vaccines, the bivalent, quadrivalent, and 9-valent HPV vaccine formulations, have been approved for use in many countries [11], and it is estimated that increased vaccine coverage will greatly accelerate the decline in incidence of and death from cervical cancer [12].

In conclusion, cervical cancer epidemiological patterns have changed dramatically over recent decades, emphasizing the importance of tracking changing trends in this context. Therefore, the authors estimated the global, regional, and national trends in cervical cancer from 1990 to 2019, using the data from the latest version of the Global Burden of Disease study (GBDs), to inform health care strategies.

## Methods

### Data source

The GBDs provides a methodological and conceptual framework for estimation and quantification of health loss worldwide, which facilitates the assessment of progress and challenges in disease control. In this work, the cervical cancer burden was mainly considered in terms of incidence, death, and DALYs. The subject term, ‘cervical cancer’, was explored using the Global Health Data Exchange (GHDx) query tool (http://ghdx.healthdata.org/gbd-results-tool), including the following parameters: time interval, age groups, and geographic locations. Cervical cancer burden data (rate, number of cases) were extracted according to age, sociodemographic index (SDI) areas, geographic regions, and countries/territories, from 1990 to 2019, without any inclusion/exclusion criteria. According to SDI, regions and countries were classified into five categories: low, low-middle, middle, high-middle, and high. Data were available from 21 geographic regions and 204 countries/territories worldwide. Human Development Index (HDI) data were obtained from the United Nations Development Program (http://hdr.undp.org/en /data).

### Statistical analysis

Age-standardization is a necessary when considering differences in the age structure of multiple populations over time. Age-standardized rate (ASR) was estimated using the following formula:

$$ \mathrm{ASR}=\frac{\sum_{i=1}^A{a}_i{w}_i}{\sum_{i=1}^A{w}_i}\times 100,000 $$

where, *a*_*i*_ represents the age-specific rate in the *i*^th^ age group, *w* represents the number of people (or the weight) in the respective *i*^th^ age group from among the selected standard population, and *A* represents the number of age groups.

The estimated annual percentage change (EAPC) is a widely accepted index to quantify and describe the trend in ASR [13]. A regression line was fitted to the natural logarithm of the rates (ASR). EAPC and its 95% confidence interval (CI) were estimated using a linear regression model. The formulae was as follows:

$$ \mathrm{y}=\upalpha +\upbeta \mathrm{x}+\upvarepsilon, $$

$$ \mathrm{EAPC}=100\times \left(\exp \left(\upbeta \right)-1\right) $$

where y = ln (ASR) and x = calendar year. Trends were assessed as follows: 1. EAPC and its 95% CI > 0 signified an increasing trend in ASR; 2. EAPC value and its 95% CI < 0 signified a decreasing trend in ASR; 3. Other outcomes signified that ASR was stable over time. To explore the impact factors of EAPC, the relationship between EAPCs values and ASR in 1990, and between EAPC values and HDI in 2019, were assessed using the Pearson correlation analysis. Data were analyzed using R program (Lucent Technologies, Jasmine Mountain, USA; Version 3.6.2). A *p* value of < 0.05 was considered to be statistically significant.

## Results

### Trends in the incidence of cervical cancer

**Fig. 1:**
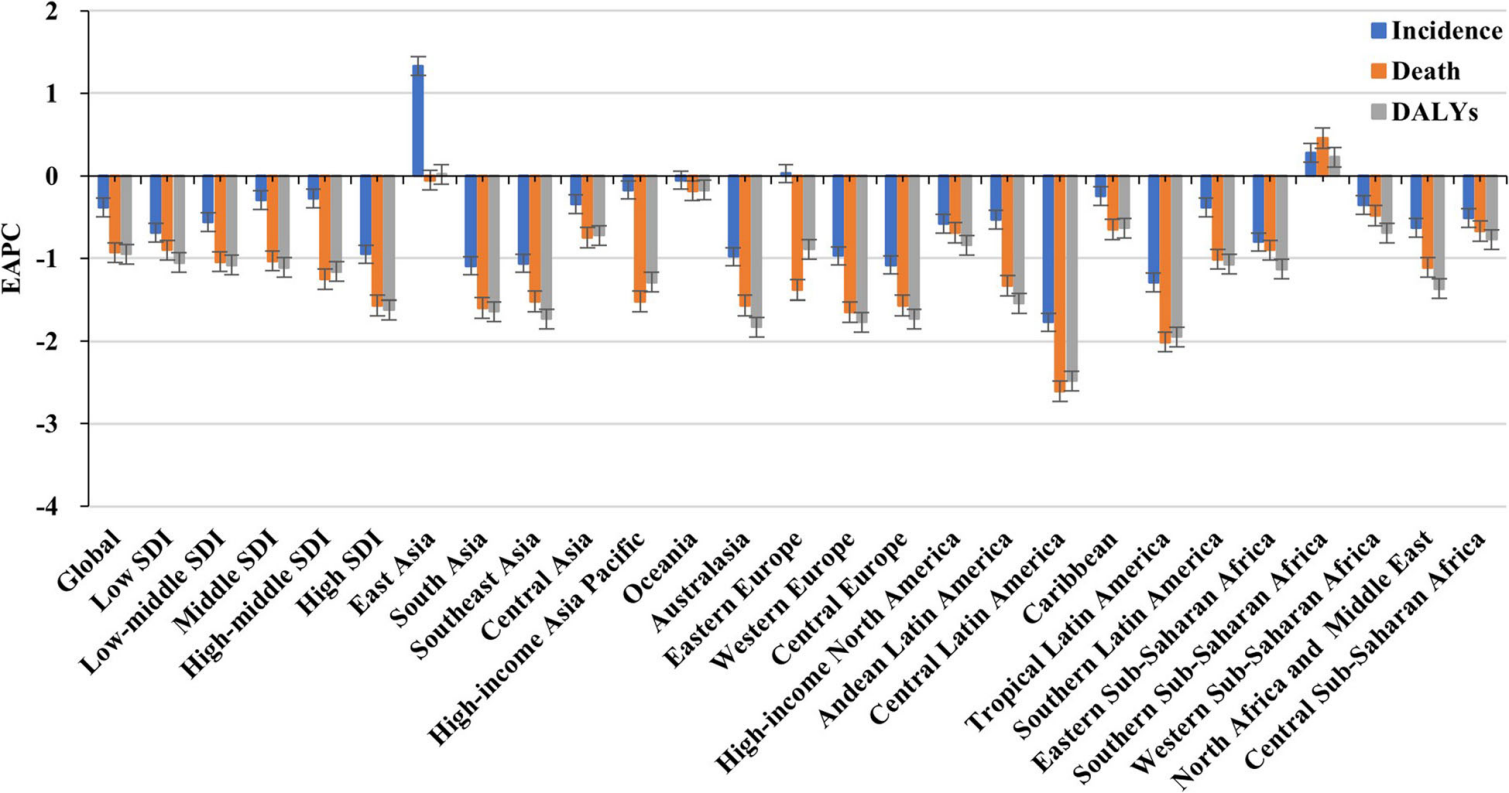
The trends in incidence, death, and DALYs of cervical cancer globally, and in SDI areas and geographic regions from 1990 to 2019. SDI, sociodemographic index; DALYs, disability-adjusted life years

**Fig. 2:**
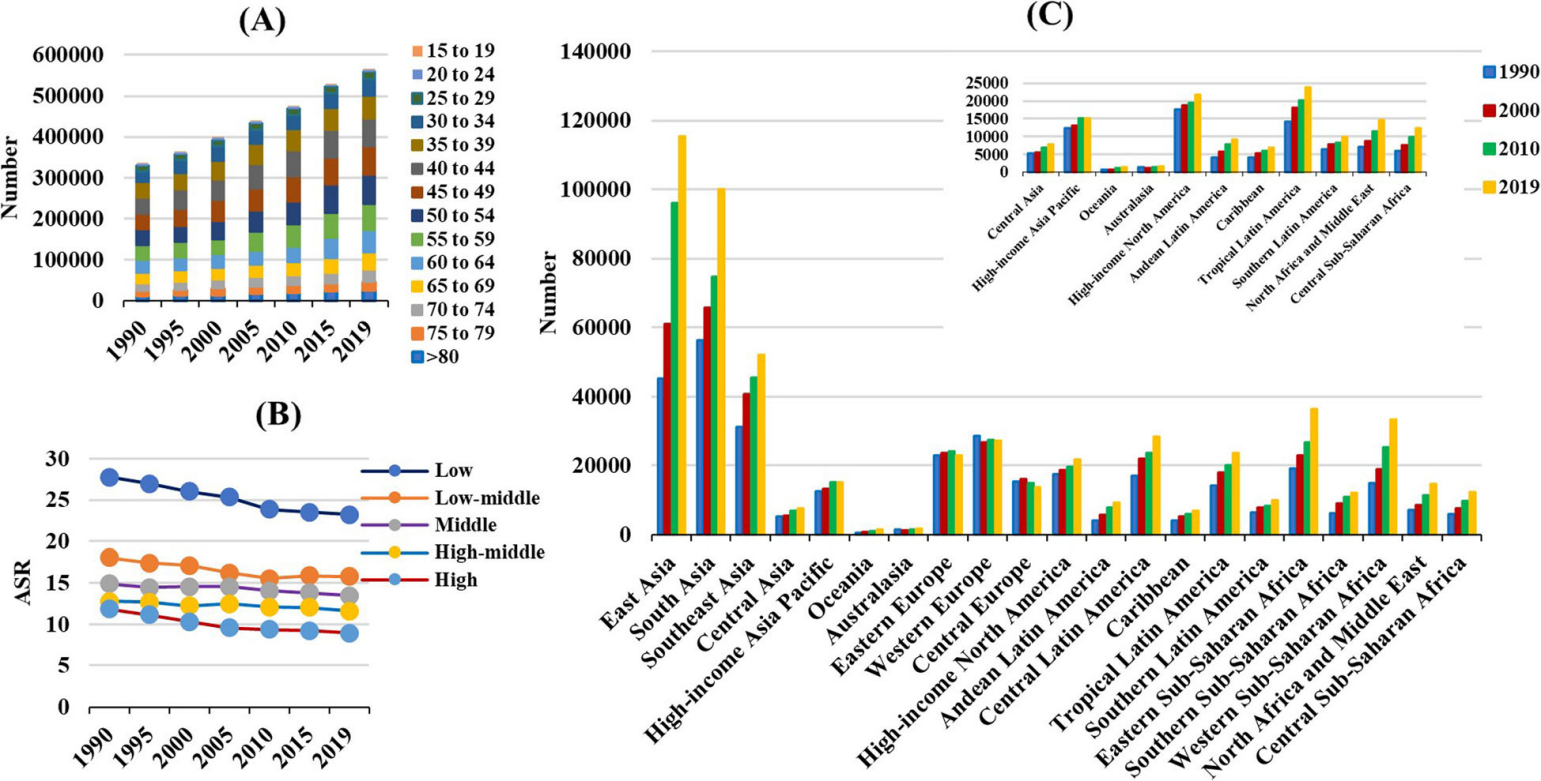
The distribution of the incident number of cervical cancer worldwide, and in SDI areas and geographic regions from 1990 to 2019. **a** the incident number in age groups; **b** the ASR in SDI areas; **c** the incident number in geographical regions. SDI, sociodemographic index; ASR, age-standardized rate

**Fig. 3:**
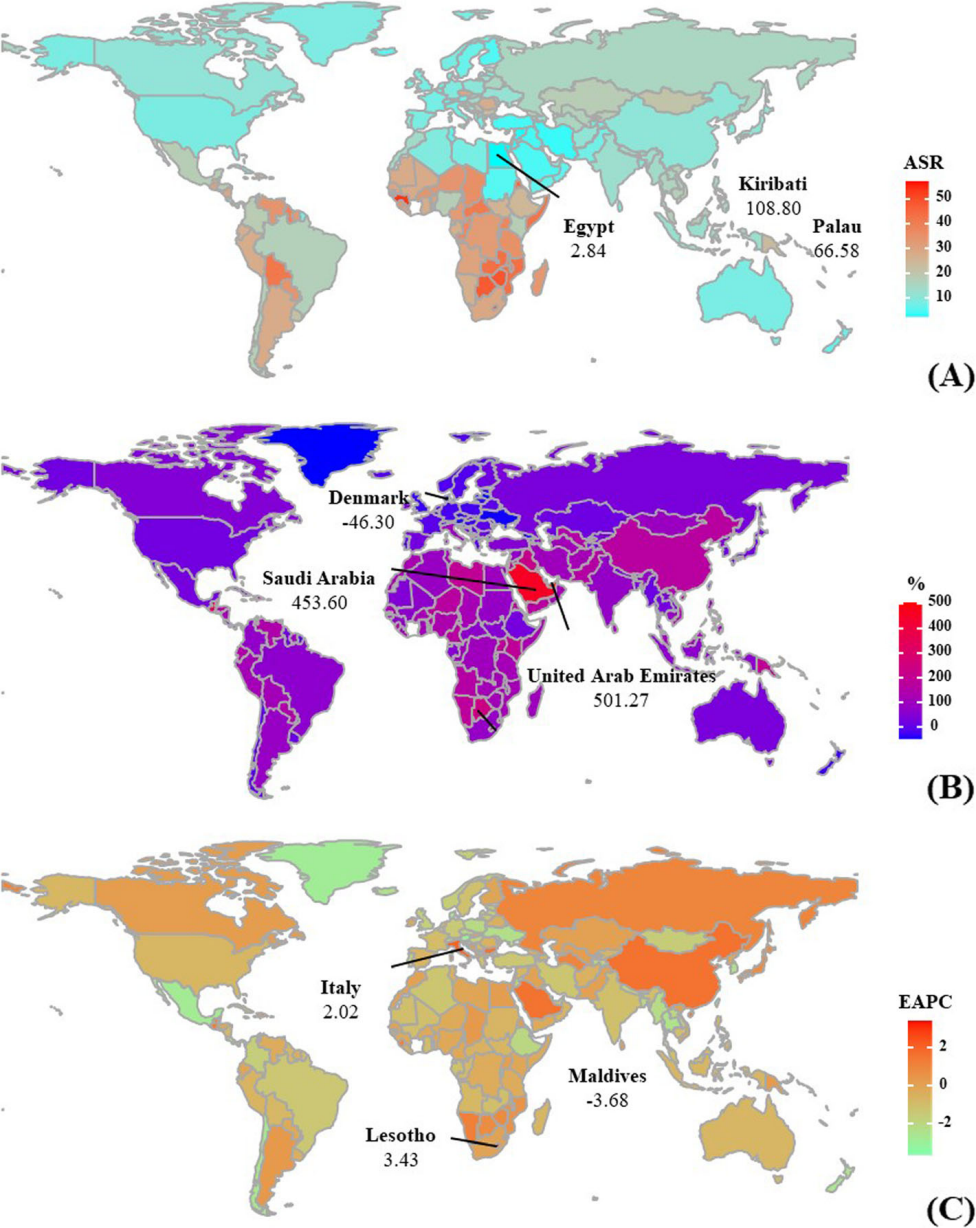
The distribution of ASR, percentage changes in number, and EAPCs of cervical cancer incidence at the national level, 1990–2019. The follows were **a** the ASR in 2019; **b** the percentage changes in number between 2000 and 2019; **c** the EAPCs in countries/territories, respectively. Countries/territories with an extreme value were annotated. ASR, age-standardized rate; EAPC, estimated annual percentage change

There were 565.54 × 10^3^ (95% uncertainty interval [UI]: 481.52 × 10^3^ to 636.43 × 10^3^) incident cases of cervical cancer in the world in 2019, representing an increase of 68.50% since 1990. The overall age-standardized incidence rate (ASIR) showed a downward trend between 1990 and 2019, decreasing by an annual average of 0.38% per year (EAPC = − 0.38; 95% CI: − 0.41 to − 0.34) (Table 1 and Fig. 1). Increasing percentage changes in number were detected in all age groups, with the highest alteration in the groups aged > 80 (118.81%) and 50–54 (90.42%) years (Supplementary Table 1 and Fig. 2 a). Meanwhile, analysis according to SDI showed decreasing trends in all areas, particularly those with high SDI (EAPC = − 0.95; 95% CI: − 1.05 to − 0.85) (Table 1 and Fig. 2 b). Among geographic regions, East Asia (115.38 × 10^3^) had the most cases in 2019. The incident trends in cervical cancer decreased in most areas, of which the most pronounced were Central Latin America (EAPC = − 1.77; 95% CI: − 1.92 to − 1.62), followed by Tropical Latin America and South Asia. Increasing trends occurred only in East Asia and Southern Sub-Saharan Africa, where EAPCs rates were 1.33 (95% CI: 1.12 to 1.55) and 0.28 (95% CI: 0.06 to 0.51), respectively (Table 1 and Fig. 2 c). Among 204 countries/territories, the ASR in 2019 varied from 108.8 per 100,000 population in Kiribati to 2.84 per 100,000 population in Egypt (Fig. 3 a). The largest increase in the incident number occurred in the United Arab Emirates (501.27%) and Saudi Arabia (453.6%). Conversely, the largest decreases were observed in Denmark (− 46.30%) and Latvia (− 45.66%). The ASIR of cervical cancer showed decreasing trends in 151 countries/territories, with the greatest decreases in the Maldives, Taiwan, and Singapore, with respective EAPCs of − 3.68 (95% CI: − 4.00 to − 3.35), − 3.63 (95% CI: − 3.96 to − 3.30), and − 3.40 (95% CI: − 3.61 to − 3.18). In contrast, increasing trends were observed in 28 countries, particularly Lesotho, Italy, and China, with respective EAPCs of 3.43 (95% CI: 2.90 to 3.95), 2.02 (95% CI: 1.70 to 2.34), and 1.61 (95% CI: 1.36 to 1.86) (Supplementary Table 2 and Fig. 3 b-c).

**Table 1:** The number and age-standardized rate of cervical cancer incidence in global, sexes, SDI areas and geographic regions in 1990 and 2019, and percentage change of absolute number and the EAPCs from 1990 to 2019

Characteristics	1990		2019		1990–2019	
	Number ×10^3^ (95% UI)	ASR/100,000) (95% UI)	Number ×10^3^ (95% UI)	ASR/100,000) (95% UI)	Change innumber (%)	EAPC(95%CI)
**Overall**	335.64 (300.35–393.89)	14.91 (13.37–17.55)	565.54 (481.52–636.43)	13.35 (11.37–15.03)	68.50	− 0.38(− 0.41–-0.34)
**SDI**						
Low	41.50 (31.77–50.80)	27.74 (21.56–34.25)	78.82 (61.61–97.93)	23.21 (18.31–28.76)	89.94	−0.69(− 0.73–-0.65)
Low-middle	66.22 (54.06–81.76)	18.04 (14.86–22.49)	125.96 (107.88–150.11)	15.78 (13.57–18.87)	90.23	−0.56(− 0.65–-0.47)
Middle	92.18 (81.45–116.40)	14.87 (13.17–18.85)	183.34 (144.49–208.86)	13.44 (10.61–15.28)	98.89	−0.29(− 0.33–-0.24)
High-middle	75.80 (71.53–88.88)	12.77 (12.05–15)	113.12 (89.78–129.15)	11.59 (9.18–13.24)	49.23	−0.27(− 0.31–-0.22)
High	59.69 (54.3–61.65)	11.83 (10.67–12.22)	63.86 (55.71–71.45)	8.91 (7.74–9.99)	6.99	−0.95(−1.05–-0.85)
**Regions**						
East Asia	45.26 (35.38–79.36)	9.00 (7.08–15.63)	115.38 (64.35–147.12)	11.17 (6.25–14.26)	154.94	1.33 (1.12–1.55)
South Asia	56.36 (44.21–68.59)	16.04 (12.64–19.66)	100.02 (80.11–124.77)	12.37 (9.94–15.46)	77.48	−1.09(−1.29–-0.90)
Southeast Asia	31.13 (23.52–38.68)	18.75 (14.3–23.63)	52.06 (41.93–68.67)	14.48 (11.73–19)	67.26	−1.06(−1.17–-0.96)
Central Asia	5.27 (4.9–5.63)	18.58 (17.37–19.85)	7.67 (6.65–8.83)	16.00 (13.94–18.4)	45.34	−0.34(− 0.45–-0.23)
High-income Asia Pacific	12.47 (11.64–14.36)	11.65 (10.81–13.42)	15.06 (11.91–17.96)	10.33 (7.99–12.4)	20.82	−0.17(− 0.27–-0.06)
Oceania	0.57 (0.4–0.76)	29.58 (21.39–39.83)	1.33 (0.86–1.82)	28.22 (19–38.09)	133.17	−0.05(− 0.12–0.03)
Australasia	1.37 (1.15–1.47)	11.83 (9.76–12.65)	1.65 (1.27–2.11)	8.22 (6.32–10.59)	20.06	−0.98(−1.36–-0.6)
Eastern Europe	22.82 (19.67–24.65)	14.53 (12.66–15.79)	23.00 (18.91–28.03)	14.76 (11.91–18.14)	0.77	0.03(−0.12–0.18)
Western Europe	28.60 (25.91–29.68)	11.19 (9.88–11.62)	27.17 (22.69–31.7)	8.26 (6.85–9.68)	−4.99	−0.97(−1.07–-0.88)
Central Europe	15.39 (14.39–16.21)	20.67 (19.23–21.74)	13.68 (11.26–15.9)	15.80 (12.97–18.48)	−11.14	−1.08(− 1.21–-0.96)
High-income North America	17.53 (15.11–18.26)	10.39 (8.89–10.83)	21.85 (17.42–26.62)	8.93 (7.09–10.93)	24.65	−0.58(−0.71–-0.44)
Andean Latin America	4.10 (3.45–4.86)	33.39 (28.20–39.63)	9.10 (6.93–11.61)	29.74 (22.67–37.83)	121.97	−0.53(−0.66–-0.41)
Central Latin America	17.08 (15.8–17.85)	32.30 (29.43–33.73)	28.48 (23.11–35.03)	21.45 (17.44–26.37)	66.74	−1.77(−1.92–-1.62)
Caribbean	4.12 (3.33–4.72)	28.00 (22.69–31.86)	6.86 (5.36–8.50)	26.23 (20.41–32.58)	66.57	−0.24(−0.30–-0.19)
Tropical Latin America	14.12 (13.36–16.36)	24.52 (23.12–28.28)	23.74 (22.13–27.18)	17.91 (16.69–20.43)	68.15	−1.29(−1.39–-1.19)
Southern Latin America	6.48 (6.05–6.87)	26.30 (24.53–27.91)	9.84 (7.27–12.85)	24.85 (18.23–32.74)	51.93	−0.38(−0.52–-0.25)
Eastern Sub-Saharan Africa	19.08 (14.41–23.81)	38.27 (28.81–47.55)	36.33 (25.76–48.45)	31.79 (22.9–41.68)	90.43	−0.80(−0.87–-0.73)
Southern Sub-Saharan Africa	6.17 (4.68–7.53)	33.33 (25.19–40.62)	12.02 (9.74–14.44)	32.90 (26.88–39.48)	94.80	0.28 (0.06–0.51)
Western Sub-Saharan Africa	14.85 (11.66–18.64)	28.64 (22.59–35.8)	33.37 (26.14–42.54)	25.47 (20.17–31.94)	124.75	−0.35(−0.40–-0.31)
North Africa and Middle East	7.003 (5.03–8.03)	6.90 (4.92–7.88)	14.63 (11.14–17.63)	5.78 (4.43–6.89)	107.98	−0.63(−0.72–-0.54)
Central Sub-Saharan Africa	5.84 (3.95–7.83)	37.38 (25.91–49.42)	12.30 (8.23–16.88)	32.32 (21.74–44.74)	110.47	−0.51(− 0.61–-0.41)

*EAPC* Estimated annual percentage change, *ASR* Age-standardized rate, *CI* Confidence interval, *UI* Uncertainty interval, *SDI* Socio-demographic index. Percentage change in absolute number was calculated based on the crew data

### Trends in death caused by cervical cancer

The overall number of deaths caused by cervical cancer was 184.53 × 10^3^ (95% UI: 164.84 × 10^3^ to 218.94 × 10^3^) in 2019, representing a 52.00% increase since 1990. The age-standardized death rate (ASDR) showed a decreasing trend from 1990 to 2019, with an annual average reduction of 0.93% per year (EAPC = − 0.93; 95% CI: − 0.98 to − 0.88) (Supplementary Table 3 and Fig. 1). Decreasing percentage changes were found in younger age groups, with the highest in the 15–19-year-old age group (− 23.71%) (Supplementary Table 1 and Supplementary Figure 1A). The ASDR showed a downward trend in all SDI areas, particularly high SDI areas (EAPC = − 1.57; 95% CI: − 1.68 to − 1.46) (Supplementary Table 3 and Supplementary Figure 1B). Geographically, the largest number of deaths was observed in South Asia (1833.69 × 10^3^) in 2019. Downward trends were observed in most regions, particularly Central and Latin America (EAPC = − 2.61; 95% CI: − 2.76 to − 2.46), followed by Tropical Latin America and Western Europe. Increasing trends only occurred in Southern Sub-Saharan Africa, in which EAPCs were 0.46 (95% CI: 0.19 to 0.72) (Supplementary Table 3 and Supplementary Figure 1C). Among 204 countries/territories, the highest ASR in 2019 occurred in Kiribati (69.52 per 100,000 population) and the lowest in Kuwait (1.76 per 100,000 population) (Supplementary Figure 3A). The most significant increases in numbers of deaths were noted in the United Arab Emirates (349.67%) and Guatemala (276.37%). In contrast, the largest decreases were observed in the Ukraine (− 50.7%) and Denmark (− 48.58%). Decreasing trends in ASDR were detected in 174 countries/territories, particularly the Maldives, Taiwan, and Singapore, in which the respective EAPCs were − 4.54 (95% CI: − 4.86 to − 4.22), − 4.13 (95% CI: − 4.44 to − 3.82), and − 4.04(95% CI: − 4.26 to − 3.81). Conversely, increasing trends were observed in 30 countries, notably Lesotho, Zimbabwe, and Bulgaria, with respective EAPCs of 3.25 (95% CI: 2.72 to 3.77), 1.46 (95% CI: 0.95 to 1.97), and 1.20 (95% CI: 0.92 to 1.48) (Supplementary Table 4 and Supplementary Figure 3B-C).

### Trends in DALYs caused by cervical cancer

During the period 1990–2019, the number of DALYs caused by cervical cancer increased by 44.99% to 8955.01 × 10^3^ (95% UI: 7547.73 × 10^3^ to 9978.46 × 10^3^) in 2019. The ASR of DALYs had a downward trend from 1990 to 2019, with an annual average of 0.95% (EAPC = − 0.95; 95% CI: − 1.00 to − 0.90) (Supplementary Table 5 and Fig. 1). There were decreasing percentage changes in DALYs in younger age groups, with largest in the 15–19-year-old age group (− 22.89%) (Supplementary Table 1 and Supplementary Figure 2A). DALYs attributable to cervical cancer showed decreasing trends in all SDI areas, and particularly high SDI areas (EAPC = − 1.62; 95% CI: − 1.74 to − 1.49) (Supplementary Table 5 and Supplementary Figure 2B). At the regional level in 2019, the greatest number of DALYs was in South Asia (1833.69 × 10^3^) The ASR of DALYs showed decreasing trends in most regions, particularly Central Latin America (EAPC = − 2.48; 95% CI: − 2.63 to − 2.32), followed by Tropical Latin America (EAPC = − 1.95; 95% CI: − 2.05 to − 1.85) and Australasia (EAPC = − 1.83; 95% CI: − 2.26 to − 1.41). Increasing trends occurred only in East Asia and Southern Sub-Saharan Africa, with respective EAPCs of 0.02 (95% CI: − 0.20 to 0.24) and 0.23 (95% CI: − 0.04 to 0.51) (Supplementary Table 5 and Supplementary Figure 2C). The country with the highest ASR in 2019 was Kiribati (2143.06 per 100,000 population), and that with the lowest was Kuwait (44.34 per 100,000 population) (Supplementary Figure 4A). The largest increases in DALYs were observed in the United Arab Emirates (409.36%), Qatar (264.9%), and Guatemala (264.9%). Conversely, the largest decreases were in Denmark (− 46.30%), Latvia (− 45.66%), and Ukraine (− 50.22%). In general, DALYs attributable to cervical cancer were decreasing in 177 countries, with the most obvious downward trend in the Maldives (EAPC = − 5.06; 95% CI: − 5.4 to − 4.72), followed by Taiwan (EAPC = − 4.43; 95% CI: − 4.76 to − 4.11), and Singapore (EAPC = − 4.31; 95% CI: − 4.55 to − 4.07). In contrast, increasing trends were observed in 27 countries, specifically Lesotho, Zimbabwe, and Bulgaria, with respective EAPCs of 3.44 (95% CI: 2.87 to 4.02), 1.67 (95% CI: 1.09 to 2.26), and 1.23 (95% CI: 0.95 to 1.51) (Supplementary Table 6 and Supplementary Figure 4B-C).

### Analysis of factors influencing EAPC

**Fig. 4:**
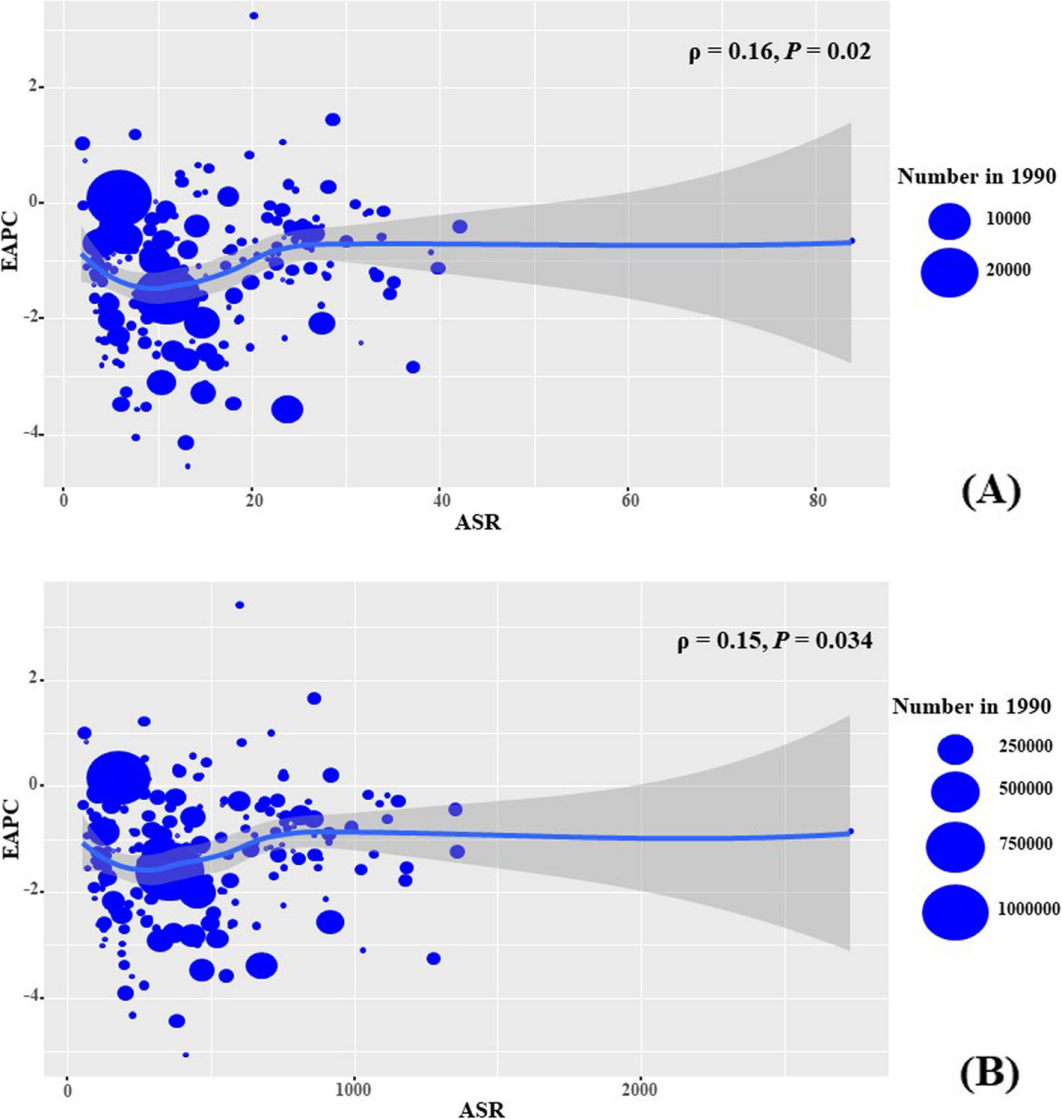
The correlation between EAPCs and ASR in 1990 at the national level. The EAPCs of death (**a**), and DALYs (**b**) had positive associations with ASR in 1990, respectively. The association was calculated with Pearson correlation analysis. The size of circle is increased with the numbers in 1990. EAPC, estimated annual percentage change; ASR, age-standardized rate. DALYs, disability-adjusted life years

**Fig. 5:**
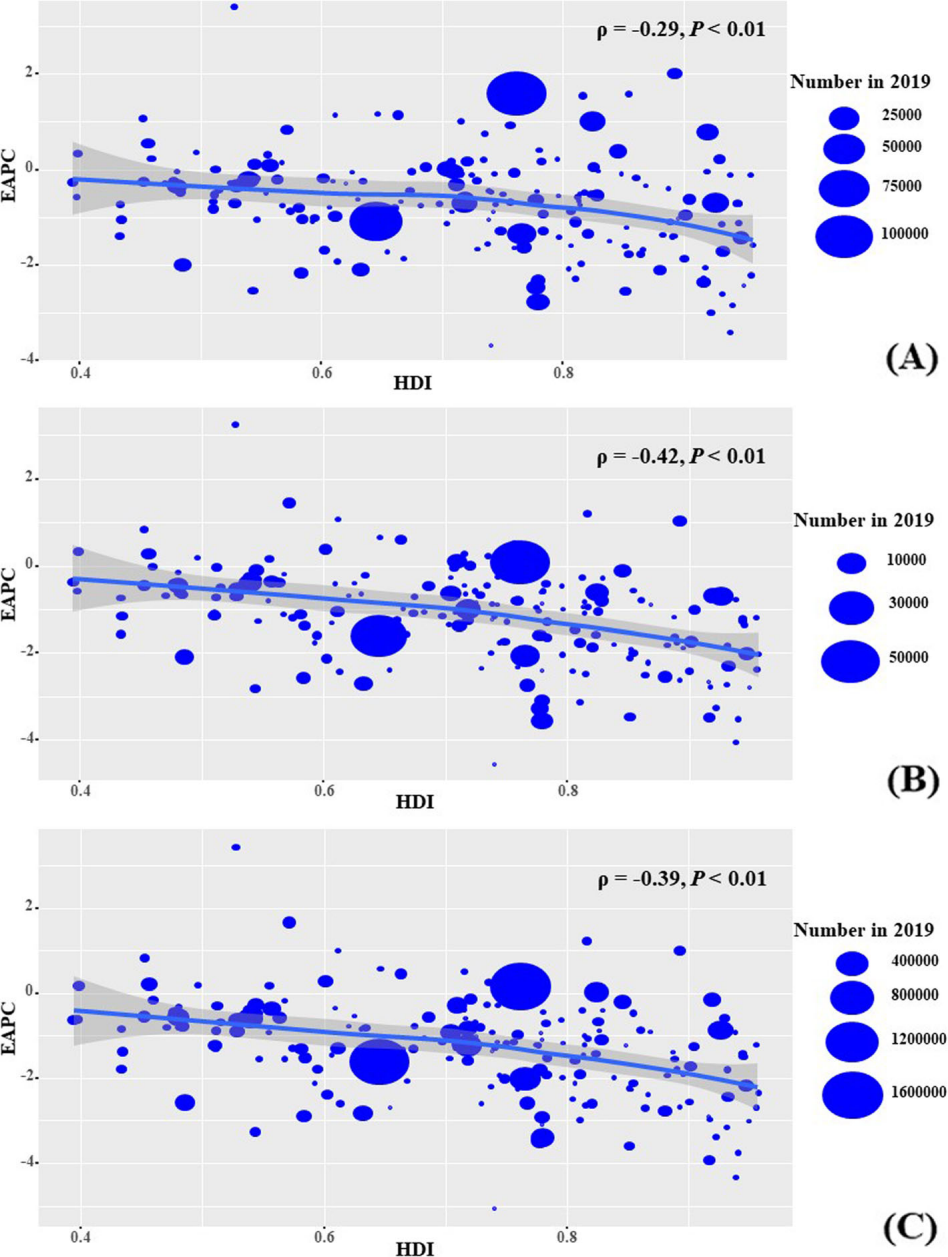
The correlation between EAPCs and HDI in 2019 at the national level. The EAPCs of incidence (**a**), death (**b**), and DALYs (**c**) had negative associations with HDI in 2019, respectively. The association was calculated with Pearson correlation analysis. The size of circle is increased with the numbers in 2019. EAPC, estimated annual percentage change; ASR, age-standardized rate. DALYs, disability-adjusted life years

During the period 1990–2019, EAPCs were positively associated with ASR death and DALYs attributable to cervical cancer (*ρ* = 0.16, *P* = 0.02; *ρ* = 0.15, *P* = 0.034, respectively) (Fig. 4 a-b). Further, EAPCs were negatively associated with HDI in 2019 in incidence, death, and DALYs attributable to cervical cancer (*ρ* = − 0.29, *P* < 0.001; *ρ* = 0.42, *P* < 0.001; *ρ* = 0.39, *p* < 0.001, respectively) (Fig. 5 a-c).

## Discussion

Decreasing trends in cervical cancer were observed worldwide from 1990 to 2019. The declining trends were predominantly a result of effective precautionary procedures combined with a series of sociocultural factors, including access to health care, changes in marriage age and family planning behavior, and enhancements in education [14].

Cervical cancer occurred across a range of ages; our data showed that the 50–54 years age group had the largest number of cases, suggesting infection at a younger age and slow progression to cancer [15]. The decreasing incidence rate of cervical cancer in young women is attributable to increasing coverage of vaccination against common subtypes of HPV [16]; however, the decreasing trends were slow, which was probably related to behavioral factors, including smoking, use of oral contraception, and promiscuous sexual behaviors [17–19].

Cervical cancer is the malignancy with the largest inter-country range of variation in mortality among all cancers [20]. Morbidity of cervical cancer is closely related to socioeconomic level; it was the highest in developing countries (e.g. Southern Sub-Saharan Africa) in 2019, which can be explained by limitations in knowledge, screening services, and sexual behavior [21, 22]. In contrast, the lowest cervical cancer ASIR was observed in North Africa and the Middle East, which may be due to cultural factors and conservative sexual behaviors [23]. These regions also have low rates of other sexually related infections, such as HIV [24]. In general, decreasing trends in the incidence of cervical cancer were observed in most countries, with the most obvious downward trend s in the Maldives, Taiwan, and Singapore, due to cancer screening and HPV programs financed by huge government expenditure. The Maldives, Taiwan and Singapore launched national screening programs in 2014, 2004 and 1995, respectively, which greatly reduced the risk of cervical cancer [25–27]. However, increasing trends still appeared in some countries, particularly Lesotho, Italy, and China. Poverty, weak health systems, and low education levels are obstacles in Lesotho [28]. China has the largest population worldwide, and also had the highest incidence, mortality and DALYs from cervical cancer [29], which may reflect a deficiency in Pap testing in China [30]. Due to the overload of public immunization systems, vaccination coverage in 15-year-old girls has decreased in Italy over recent years [31]. Further, an online survey reported that Italian undergraduates had poor knowledge about HPV [32].

The GBD study conducted high quality assessment of cervical cancer based on good quality and quantity of data; however, this paper has some limitations: (1) vital registration and cancer registry data are poor and heterogeneous in many low-income regions, due to different screening methods, which may lead to underestimation bias in these cancer registries [33]; and (2) when actual disease burden data was not available, GBD uncertainty estimates were used to fills the gaps. In addition, this type of analysis is inevitably influenced by differences in data collection and coding as well as data source quality [34].

## Conclusions

Cervical incidence, death and DALYs showed decreasing trends at the global, regional, and national levels from 1990 to 2019; however, the trends were relatively slow, and there were large regional imbalance. Consequently, cervical cancer remains a major public health problem, and development of more effective prevention and management strategies is warranted.

## Supplementary Information

**Additional file 1: Supplementary Figure 1.** The distribution of death number of cervical cancer worldwide, and in SDI areas and geographic regions from 1990 to 2019.

**Additional file 2: Supplementary Figure 2.** The distribution of DALYs number of cervical cancer worldwide, and in SDI areas and geographic regions from 1990 to 2019.

**Additional file 3: Supplementary Figure 3.** The distribution of ASR, percentage changes in number, and EAPCs of death caused by cervical cancer at the national level, 1990-2019.

**Additional file 4: Supplementary Figure 4.** The distribution of ASR, percentage changes in number, and EAPCs of DALYs caused by cervical cancer at the national level, 1990-2019.

**Additional file 5: Supplementary Table 1.** the number of cervical cancer in 2019, and the percentage changes in number during the period 1990-2019 in age groups.

**Additional file 6: Supplementary Table 2.** the number and age–standardized rate of cervical cancer incidence at national level and both sexes in 1990 and 2019, and the percentage changes in number and the EAPCs from 1990 to 2019.

**Additional file 7: Supplementary Table 3.** The number and age-standardized rate of death due to cervical cancer in global, sexes, SDI areas and geographic regions in 1990 and 2019, and percentage change of absolute number and the EAPCs from 1990 to 2019.

**Additional file 8: Supplementary Table 4.** the number and age-standardized rate of death caused by cervical cancer at national level and both sexes in 1990 and 2019, and the percentage changes in number and the EAPCs from 1990 to 2019.

**Additional file 9: Supplementary Table 5.** The number and age-standardized rate of DALYs due to cervical cancer in global, sexes, SDI areas and geographic regions in 1990 and 2019, and percentage change of absolute number and the EAPCs from 1990 to 2019.

**Additional file 10: Supplementary Table 6.** the number and age-standardized rate of DALYs caused by cervical cancer at national level and both sexes in 1990 and 2019, and the percentage changes in number and the EAPCs from 1990 to 2019.

## Data Availability

The datasets generated and analysed during the current study are available in the Global Health Data Exchange (GHDx) software (http://ghdx.healthdata.org/gbd-results-tool).

## Abbreviations

GBD: Global Burden of Disease
DALYs: Disability-adjusted life years
ASR: Age-standardized rate
UI: Uncertainty interval
CI: Confidence interval
EAPC: Estimated annual percentage change
GHDx: Global Health Data Exchange
SDI: Socio-demographic index
