# Supplementary material for: Trends of cervical cancer at global, regional, and national level: data from the Global Burden of Disease study 2019
Source: BMC Public Health. 2021 May 12;21:894. doi: 10.1186/s12889-021-10907-5 (PMC8114503; doi:10.1186/s12889-021-10907-5)
Supplement: Supplementary file 1 — Additional file 1: Supplementary Figure 1. The distribution of death number of cervical cancer worldwide, and in SDI areas and geographic regions from 1990 to 2019. [file 12889_2021_10907_MOESM1_ESM.doc]

**
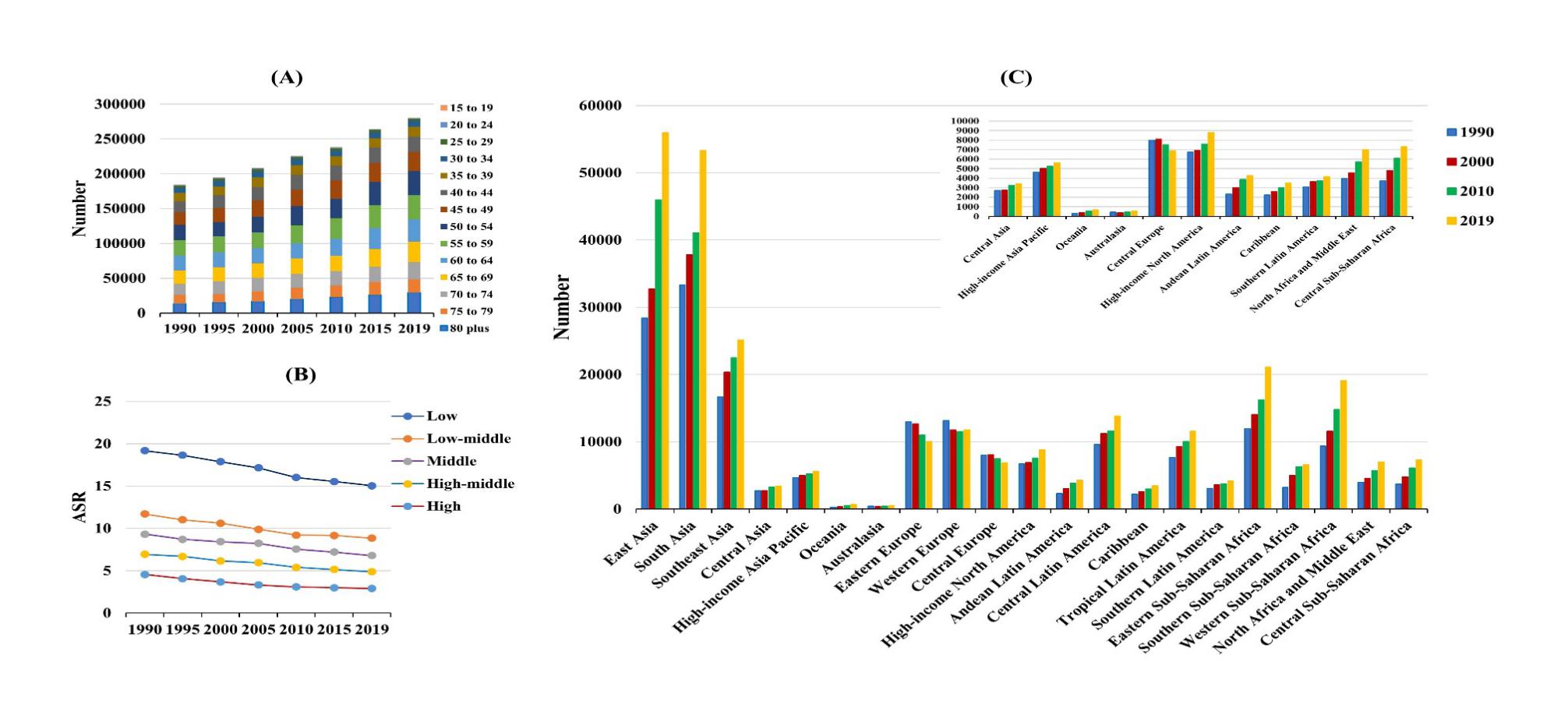
**

**Supplementary Figure 1**. The distribution of death number of cervical cancer worldwide, and in SDI areas and geographic regions from 1990 to 2019. (A) the death number in age groups; (B) the death number in SDI areas; (C) the death number in geographical regions. SDI, sociodemographic index.
