# Supplementary material for: Trends of cervical cancer at global, regional, and national level: data from the Global Burden of Disease study 2019
Source: BMC Public Health. 2021 May 12;21:894. doi: 10.1186/s12889-021-10907-5 (PMC8114503; doi:10.1186/s12889-021-10907-5)
Supplement: Supplementary file 3 — Additional file 3: Supplementary Figure 3. The distribution of ASR, percentage changes in number, and EAPCs of death caused by cervical cancer at the national level, 1990-2019. [file 12889_2021_10907_MOESM3_ESM.doc]

**
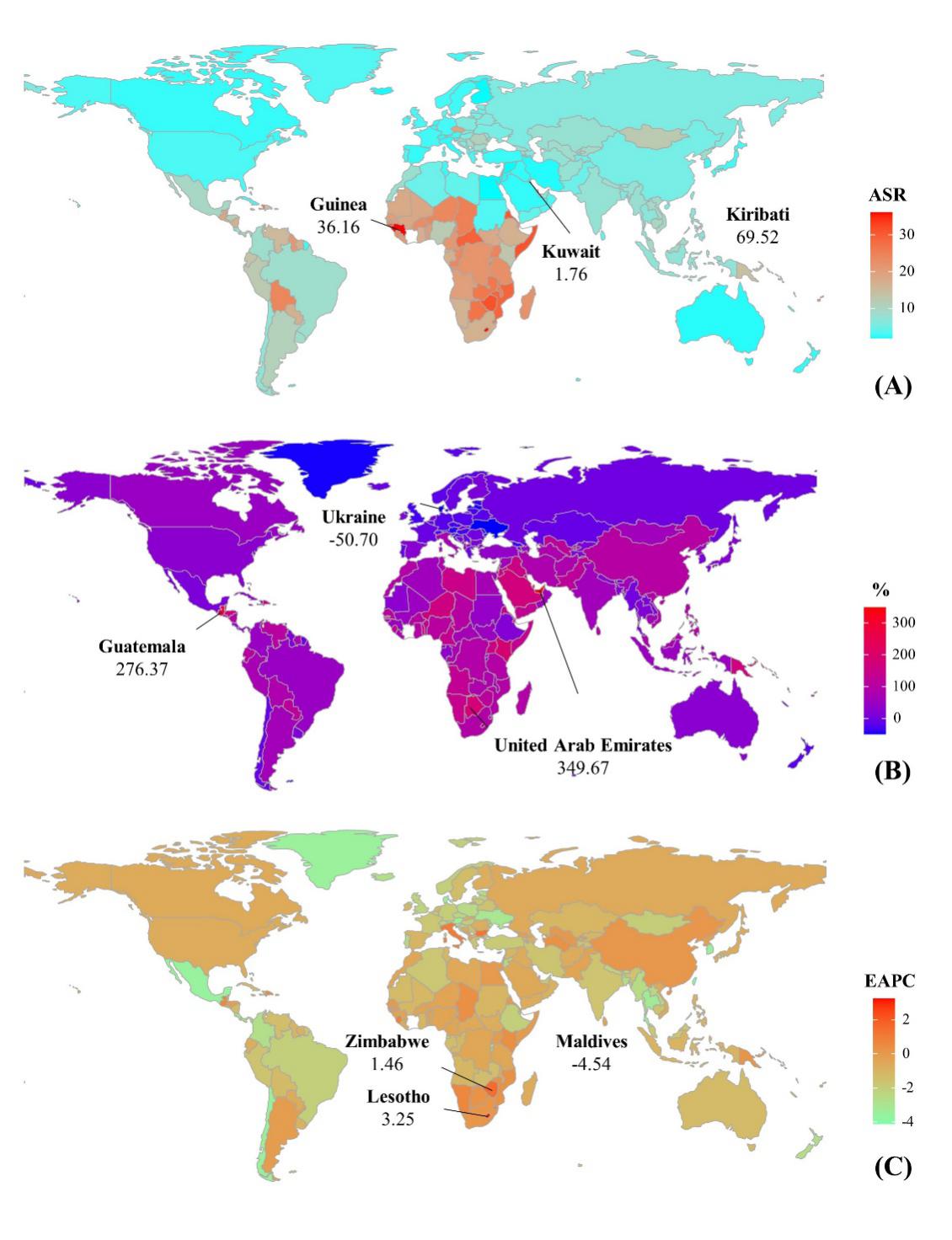
**

**Supplementary Figure 3**. The distribution of ASR, percentage changes in number, and EAPCs of death caused by cervical cancer at the national level, s1990-2019. The follows were (A) the ASR in 2019; (B) the percentage changes in number between 2000 and 2019; (C) the EAPCs in countries/territories, respectively. Countries/territories with an extreme value were annotated. ASR, age-standardized rate; EAPC, estimated annual percentage change.
