# Supplementary material for: Trends of cervical cancer at global, regional, and national level: data from the Global Burden of Disease study 2019
Source: BMC Public Health. 2021 May 12;21:894. doi: 10.1186/s12889-021-10907-5 (PMC8114503; doi:10.1186/s12889-021-10907-5)
Supplement: Supplementary file 5 — Additional file 5: Supplementary Table 1. the number of cervical cancer in 2019, and the percentage changes in number during the period 1990-2019 in age groups. [file 12889_2021_10907_MOESM5_ESM.doc]

**Supplementary Table 1**. the number of cervical cancer in 2019, and the percentage changes in number during the period 1990–2019 in age groups

| **Age Groups** | **Incidence** | | **Death** | | **DALYs** | |
| --- | --- | --- | --- | --- | --- | --- |
|  | Number  ×10^3^(95% UI) | Change in number (%) | Number  ×10^3^(95% UI) | Change in number (%) | Number  ×10^3^(95% UI) | Change in number (%) |
| **15 to 19** | 1.36(1.04–1.57) | 0.28 | 0.24(0.18–0.27) | -23.71 | 17.58(13.26–20.46) | -22.89 |
| **20 to 24** | 5.74(4.65–6.66) | 17.81 | 1.14(0.92–1.35) | -7.37 | 78.89(63.33–93.42) | -6.56 |
| **25 to 29** | 16.75(13.77–19.14) | 30.23 | 3.45(2.86–4) | 3.25 | 221.01(182.34–255.95) | 4.15 |
| **30 to 34** | 39.56(33.19–44.64) | 46.66 | 8.2(6.81–9.37) | 14.51 | 484.44(405.23–554.91) | 15.69 |
| **35 to 39** | 55.86(47.35–63.46) | 49.62 | 14.14(12.14–16.32) | 18.69 | 758.61(651.76–874.77) | 19.74 |
| **40 to 44** | 68.35(57.61–77.26) | 69.82 | 21.99(18.51–25.05) | 36.74 | 1061.38(893.68–1211.22) | 37.81 |
| **45 to 49** | 69.29(58.41–78.04) | 86.61 | 27.39(22.77–30.94) | 51.82 | 1179.32(984.77–1333.83) | 52.86 |
| **50 to 54** | 72.41(59.91–82.2) | 90.42 | 34.63(28.61–38.99) | 61.2 | 1317.79(1092.34–1480.19) | 62.09 |
| **55 to 59** | 64.99(53.94–73.06) | 78.89 | 34.91(29.18–39.22) | 55.09 | 1159.59(968.22–1301.41) | 55.84 |
| **60 to 64** | 52.61(44.84–59.39) | 65.74 | 31.73(26.85–35.8) | 48.58 | 903.28(766.17–1018.18) | 49.13 |
| **65 to 69** | 42.4(36.42–47.66) | 64.47 | 29.52(25.19–33.42) | 53.05 | 703.59(599.94–793.69) | 53.31 |
| **70 to 74** | 30.65(26.81–34.42) | 67.41 | 24.99(21.92–28.56) | 57.91 | 486.36(426.48–556.32) | 58.16 |
| **75 to 79** | 20.48(17.55–23.07) | 54.99 | 18.87(16.31–21.37) | 48.91 | 290.15(250.52–328.81) | 48.78 |
| **＞80** | 25.08(20.53–28.3) | 118.81 | 29.29(23.98–33.03) | 119.9 | 293.01(241.59–328.39) | 111.49 |

DALYs, disability-adjusted life years.
