# Supplementary material for: Trends of cervical cancer at global, regional, and national level: data from the Global Burden of Disease study 2019
Source: BMC Public Health. 2021 May 12;21:894. doi: 10.1186/s12889-021-10907-5 (PMC8114503; doi:10.1186/s12889-021-10907-5)
Supplement: Supplementary file 6 — Additional file 6: Supplementary Table 2. the number and age–standardized rate of cervical cancer incidence at national level and both sexes in 1990 and 2019, and the percentage changes in number and the EAPCs from 1990 to 2019. [file 12889_2021_10907_MOESM6_ESM.doc]

**Supplementary Table 2**. the number and age–standardized rate of cervical cancer incidence at national level and both sexes in 1990 and 2019, and the percentage changes in number and the EAPCs from 1990 to 2019

|  | **1990** | | **2019** | | **1990-2019** | |
| --- | --- | --- | --- | --- | --- | --- |
| **Characteristics** | Number  ×10^2^ (95% UI) | ASR/100,000)  (95% UI) | Number  ×10^2^ (95% UI) | ASR/100,000)  (95% UI) | Changes in number (%) | EAPCs  (95%CI) |
| Afghanistan | 4.93(1.49–7.68) | 12.79(4.02–19.73) | 10.7(3.8–16.99) | 11.39(4.58–17.47) | 116.76 | -0.52(-0.67–-0.37) |
| Albania | 1.03(0.9–1.39) | 8.06(7.07–11) | 1.4(0.97–1.94) | 7.98(5.5–11.18) | 36.02 | 0.23(0.05–0.41) |
| Algeria | 10.28(6.83–13.52) | 13.71(9.32–17.87) | 19.17(13.03–26.93) | 9.48(6.64–12.96) | 86.52 | -1.28(-1.42–-1.15) |
| American Samoa | 0.02(0.02–0.03) | 16.83(13.45–22.98) | 0.05(0.04–0.06) | 18.65(14–24.23) | 103.24 | 0.44(0.28–0.6) |
| Andorra | 0.04(0.03–0.05) | 13.41(9.44–18.58) | 0.08(0.05–0.1) | 12.49(8.68–16.96) | 92.05 | -0.33(-0.42–-0.24) |
| Angola | 10.02(6.44–14.81) | 36.67(23.69–52.94) | 25.97(16.15–38.46) | 30.31(19.32–44.76) | 159.06 | -0.8(-0.93–-0.67) |
| Antigua and Barbuda | 0.07(0.06–0.08) | 23.31(19.97–27.17) | 0.12(0.1–0.15) | 21.44(17.36–26.27) | 80.73 | -0.45(-0.58–-0.32) |
| Argentina | 39.39(36.25–43.08) | 23.71(21.77–25.97) | 72.82(51.69–96.49) | 28.02(19.75–37.24) | 84.88 | 0.39(0.23–0.56) |
| Armenia | 3.15(2.82–3.51) | 19.15(17.25–21.52) | 3.23(2.6–3.99) | 15.61(12.47–19.13) | 2.44 | -0.63(-0.88–-0.38) |
| Australia | 10.97(9.55–11.85) | 11.3(9.74–12.23) | 14.46(10.71–18.95) | 8.55(6.36–11.27) | 31.9 | -0.7(-1.05–-0.34) |
| Austria | 8.73(7.05–9.48) | 16.22(12.91–17.73) | 5.06(3.99–6.82) | 7.28(5.59–9.66) | -42.02 | -2.98(-3.2–-2.75) |
| Azerbaijan | 4.4(3.73–5.31) | 13.94(11.94–17.05) | 7.05(5.18–9.87) | 11.88(8.77–16.66) | 60.14 | -0.67(-0.77–-0.57) |
| Bahamas | 0.3(0.26–0.34) | 27.98(24.24–31.69) | 0.54(0.41–0.7) | 23.82(18.16–30.8) | 81.25 | -0.74(-0.85–-0.63) |
| Bahrain | 0.08(0.07–0.11) | 7.8(6.35–10.13) | 0.29(0.22–0.39) | 5.65(4.29–7.4) | 244.5 | -1.21(-1.42–-1.01) |
| Bangladesh | 53.71(23.93–70.45) | 19.35(8.73–25.15) | 74.07(40.71–115.33) | 10.07(5.5–15.46) | 37.92 | -2.09(-2.35–-1.82) |
| Barbados | 0.46(0.41–0.51) | 32.55(28.68–36.2) | 0.62(0.49–0.77) | 27.97(22.26–34.78) | 33.48 | -0.5(-0.55–-0.44) |
| Belarus | 11.39(10.48–12.56) | 16.42(15.08–18.13) | 11.15(8.19–15.51) | 15.41(11.18–21.48) | -2.1 | -0.5(-0.65–-0.36) |
| Belgium | 7.03(6.06–7.65) | 10.27(9.05–11.29) | 6.31(4.76–8.1) | 7.49(5.58–9.75) | -10.2 | -1.14(-1.25–-1.04) |
| Belize | 0.17(0.15–0.2) | 33.57(29.43–38.55) | 0.63(0.51–0.75) | 35.78(29.22–42.55) | 264.24 | -0.08(-0.47–0.3) |
| Benin | 4.08(3.13–5.4) | 32.25(25.53–42.38) | 10.24(7.01–14.71) | 30.15(21.48–42.28) | 151.28 | -0.17(-0.22–-0.12) |
| Bermuda | 0.05(0.05–0.06) | 15.04(12.85–17.45) | 0.04(0.03–0.06) | 8.34(6.44–10.74) | -17.39 | -2.57(-2.79–-2.36) |
| Bhutan | 0.32(0.15–0.46) | 20.04(9.16–28.38) | 0.41(0.24–0.66) | 12.94(7.61–20.35) | 27.92 | -1.72(-1.97–-1.47) |
| Bolivia | 9.72(6.17–12.38) | 49.06(31.72–62.24) | 20.7(14.35–28.58) | 41.59(29.32–57.22) | 113.06 | -0.77(-0.89–-0.66) |
| Bosnia and Herzegovina | 2.98(2.61–3.58) | 12.2(10.74–14.72) | 3.36(2.26–4.39) | 13.47(8.94–18) | 12.8 | 0.42(0.25–0.59) |
| Botswana | 1.42(0.89–2.19) | 37.72(24.27–57.1) | 4.76(2.72–7.47) | 47.63(28.09–73.79) | 236.2 | 0.77(0.53–1) |
| Brazil | 136.71(129.22–158.84) | 24.32(22.91–28.18) | 226.51(211.42–263.01) | 17.51(16.34–20.26) | 65.68 | -1.34(-1.43–-1.24) |
| Brunei Darussalam | 0.3(0.21–0.38) | 38.78(28.48–48.5) | 0.53(0.4–0.7) | 25.04(19.58–32.57) | 77.54 | -1.49(-1.72–-1.26) |
| Bulgaria | 10.24(9.06–11.55) | 18.6(16.14–20.8) | 11.64(7.77–15.25) | 22.94(14.44–30.39) | 13.62 | 1.56(1.29–1.84) |
| Burkina Faso | 10.22(7.4–13.69) | 36.74(27.05–49.48) | 22.75(16.2–30.34) | 34.46(25.14–45.25) | 122.72 | -0.25(-0.41–-0.1) |
| Burundi | 7.88(5.03–10.94) | 50.06(32.65–69.32) | 11.71(7.17–17.86) | 38.08(23.51–56.86) | 48.66 | -1.39(-1.56–-1.22) |
| Cabo Verde | 0.39(0.32–0.48) | 30.03(24.71–38.37) | 0.58(0.46–0.87) | 23.02(18.09–33.58) | 49.07 | -0.67(-0.99–-0.35) |
| Cambodia | 7.4(4.12–10.54) | 22.72(12.85–32.33) | 13.78(9.72–20.73) | 17.82(12.77–27.14) | 86.11 | -1.01(-1.11–-0.9) |
| Cameroon | 10.21(8–13.74) | 35.11(27.68–46.53) | 27.33(16.79–41.75) | 32.57(20.76–48.61) | 167.79 | -0.2(-0.35–-0.04) |
| Canada | 18.66(16.98–20.49) | 11.38(10.34–12.53) | 27.33(20.47–35.88) | 11.26(8.25–14.94) | 46.46 | 0.22(0.07–0.38) |
| Central African Republic | 3.84(2.46–5.28) | 47.14(30.65–64.29) | 6.5(3.76–9.73) | 40.68(24.48–61.06) | 69.4 | -0.56(-0.69–-0.43) |
| Chad | 5.67(4.32–7.8) | 33.47(25.62–46.44) | 12.51(8.43–16.82) | 35.47(24.31–47.3) | 120.45 | 0.34(0.24–0.45) |
| Chile | 21.21(18.48–22.85) | 35.29(30.48–37.92) | 20.8(15.49–27.41) | 18.08(13.36–24.15) | -1.93 | -2.54(-2.75–-2.33) |
| China | 406.81(309.2–731.82) | 8.41(6.44–15) | 1097.6(581.89–1415.39) | 11.01(5.87–14.22) | 169.81 | 1.61(1.36–1.86) |
| Colombia | 29.71(27.53–33.12) | 26.46(24.61–29.32) | 51.54(38.52–67.32) | 18.73(13.99–24.45) | 73.46 | -1.63(-1.83–-1.42) |
| Comoros | 0.5(0.2–0.77) | 37.65(16.69–57.37) | 1.03(0.63–1.56) | 34.71(21.85–51.81) | 105.46 | -0.48(-0.69–-0.26) |
| Congo | 3.39(2.17–4.61) | 47.76(32.24–64.15) | 6.93(4.3–10.37) | 37.24(23.63–54.19) | 104.4 | -0.86(-1.03–-0.7) |
| Cook Islands | 0.01(0.01–0.01) | 12.13(8.82–16.82) | 0.01(0.01–0.01) | 8.86(5.87–11.89) | 21.16 | -0.73(-1.06–-0.4) |
| Costa Rica | 3.03(2.62–3.31) | 28.16(24.41–30.66) | 4.6(3.43–6.18) | 16.63(12.37–22.34) | 51.76 | -2.27(-2.67–-1.87) |
| Croatia | 6.62(5.46–7.46) | 20.12(16.92–22.69) | 4.25(3.18–5.58) | 12.25(9–16.24) | -35.8 | -1.6(-1.94–-1.26) |
| Cuba | 12.6(11.05–13.76) | 23.6(20.64–25.75) | 15.53(11.96–19.31) | 19.25(14.65–24.19) | 23.29 | -0.92(-1.1–-0.75) |
| Cyprus | 0.32(0.25–0.46) | 7.61(6.06–10.98) | 0.61(0.42–0.74) | 6.49(4.56–8) | 92.98 | -0.39(-0.56–-0.22) |
| Czechia | 12.47(11.55–13.36) | 18.73(17.28–20.18) | 8.98(7.18–11.27) | 11.2(8.86–14.28) | -27.97 | -1.87(-1.94–-1.79) |
| Côte d'Ivoire | 8.7(6.44–11.5) | 31.4(23.99–40.64) | 20.34(13.37–29) | 28.24(19.14–39.64) | 133.9 | -0.15(-0.26–-0.03) |
| Democratic People's Republic of Korea | 21.06(13.59–35.88) | 19.77(13.05–33.65) | 31.46(19.92–46.73) | 18.46(11.61–27.49) | 49.35 | -0.08(-0.18–0.02) |
| Democratic Republic of the Congo | 39.5(26.35–53.65) | 36.16(24.54–48.68) | 80.68(52.09–112.95) | 32.31(21.05–45.37) | 104.28 | -0.35(-0.45–-0.25) |
| Denmark | 6.79(5.33–7.3) | 19.71(15.3–21.33) | 3.65(2.75–5.04) | 8.98(6.63–12.41) | -46.3 | -2.84(-3.15–-2.54) |
| Djibouti | 0.38(0.22–0.57) | 36.34(22.28–52.87) | 1.39(0.76–2.5) | 33.9(19.41–59.47) | 266.15 | -0.27(-0.35–-0.19) |
| Dominica | 0.14(0.12–0.17) | 41.14(33.4–49.95) | 0.13(0.1–0.17) | 33.46(25.16–43.25) | -4.33 | -0.87(-0.98–-0.77) |
| Dominican Republic | 4.69(3.89–6.11) | 19.78(16.58–25.62) | 13.12(9.14–18.61) | 25.4(17.82–36.07) | 179.97 | 0.94(0.78–1.1) |
| Ecuador | 9.09(7.76–10.5) | 28.69(24.53–33) | 22.53(16.87–30.45) | 27.2(20.37–36.61) | 147.78 | -0.06(-0.28–0.17) |
| Egypt | 4.93(4.19–6.23) | 2.86(2.44–3.69) | 9.7(6.56–14.23) | 2.84(1.94–4.13) | 96.66 | 0.19(0.09–0.28) |
| El Salvador | 4.91(4.37–6.33) | 27.41(24.46–35.27) | 9.91(7.08–13.62) | 29.33(20.92–40.42) | 101.73 | -0.43(-0.88–0.02) |
| Equatorial Guinea | 0.51(0.3–0.74) | 37.71(22.52–55.23) | 1.05(0.61–1.79) | 27.74(16.5–45.72) | 106.65 | -1.08(-1.22–-0.95) |
| Eritrea | 3.44(2.08–5.31) | 42.99(25.84–66.52) | 9.15(5.72–13.59) | 44.96(28.1–65.61) | 165.59 | 0.24(0.17–0.3) |
| Estonia | 2.53(2.2–2.76) | 23.53(20.58–25.9) | 1.69(1.25–2.2) | 16.61(12.17–21.99) | -33.37 | -1.38(-1.55–-1.22) |
| Eswatini | 0.71(0.46–1) | 34.31(22.22–48.24) | 1.66(0.83–2.84) | 41.02(20.87–70.5) | 133.83 | 1.16(0.56–1.76) |
| Ethiopia | 51.24(24.65–81.27) | 38.56(20.63–60.52) | 65.67(44.69–106.42) | 24.6(17.1–39.23) | 28.16 | -1.99(-2.19–-1.79) |
| Fiji | 1.17(0.58–1.57) | 46.1(22.97–60.94) | 1.77(0.73–2.43) | 40.44(16.93–55.32) | 50.56 | -0.08(-0.38–0.23) |
| Finland | 2.07(1.87–2.3) | 5.75(5.18–6.5) | 2.4(1.72–3.14) | 5.18(3.8–6.88) | 16.02 | -0.1(-0.22–0.03) |
| France | 40.58(34.81–43.62) | 10.89(9.44–11.71) | 38.88(29.4–50.86) | 8.1(6.02–10.65) | -4.18 | -0.94(-1.02–-0.86) |
| Gabon | 1.17(0.81–1.58) | 36.03(24.93–48.65) | 1.84(1.16–2.73) | 26.93(17.31–39.47) | 57 | -1.12(-1.36–-0.88) |
| Gambia | 0.5(0.33–0.69) | 22.04(14.96–30.35) | 1.66(1.12–2.33) | 26.1(18.07–36.18) | 234.29 | 0.36(0.13–0.58) |
| Georgia | 7.75(5.92–8.8) | 23.52(18–26.8) | 4.45(3.46–5.43) | 17.18(13.15–21.12) | -42.62 | -0.57(-1.13–-0.01) |
| Germany | 78.15(70.67–83.53) | 13.8(11.93–14.87) | 62.2(46.77–84.59) | 9.33(6.93–12.75) | -20.4 | -1.43(-1.54–-1.33) |
| Ghana | 15.36(11.22–20.9) | 35.03(26.54–47.73) | 32.33(21.5–45.3) | 27.65(18.63–37.87) | 110.43 | -0.97(-1.05–-0.88) |
| Greece | 7.53(6.77–8.2) | 11.19(9.88–12.26) | 6.89(5.28–9.02) | 8.28(6.2–11.03) | -8.55 | -1.09(-1.28–-0.89) |
| Greenland | 0.08(0.06–0.1) | 36.09(28.79–45.41) | 0.08(0.06–0.1) | 24.62(18.46–32.45) | -6.22 | -1.75(-1.92–-1.57) |
| Grenada | 0.15(0.13–0.17) | 41.28(35.53–47.95) | 0.19(0.16–0.23) | 34.28(28.06–40.41) | 33 | -0.65(-0.87–-0.43) |
| Guam | 0.07(0.06–0.09) | 15.25(12.6–18.85) | 0.12(0.09–0.16) | 13.65(10.27–17.49) | 75.49 | -0.75(-1.05–-0.44) |
| Guatemala | 5.17(4.13–9.04) | 22.2(18.01–37.81) | 22.15(16.1–29.21) | 31.18(22.27–40.9) | 328.35 | 1.15(0.63–1.68) |
| Guinea | 11.52(8.81–14.58) | 59.45(45.47–75.24) | 19.21(13.78–26.16) | 53.61(38.58–72) | 66.73 | -0.23(-0.28–-0.18) |
| Guinea-Bissau | 1.36(0.89–1.94) | 47.94(31.42–67.88) | 2.5(1.54–3.58) | 44.77(28.38–62.6) | 84.24 | -0.03(-0.13–0.06) |
| Guyana | 1.15(0.93–1.4) | 45.9(37.35–55.6) | 1.45(1.05–1.94) | 38.85(28.34–51.69) | 25.66 | -0.75(-0.95–-0.54) |
| Haiti | 12.43(4.89–16.93) | 58.11(24.4–77.15) | 22.08(10.04–33.31) | 44.12(20.18–65.83) | 77.62 | -0.82(-0.91–-0.72) |
| Honduras | 2.78(2.12–3.58) | 19.58(15.07–25.1) | 7.31(4.36–11.43) | 18.99(11.67–29) | 162.54 | -0.24(-0.38–-0.09) |
| Hungary | 14.8(13.55–16.11) | 21.48(19.47–23.46) | 10.67(8.36–13.52) | 14.27(10.96–18.31) | -27.91 | -1.77(-1.93–-1.61) |
| Iceland | 0.14(0.12–0.16) | 10.51(8.87–11.95) | 0.13(0.1–0.15) | 5.77(4.7–6.96) | -10 | -2.42(-2.58–-2.26) |
| India | 474.09(371.34–605.74) | 16.65(13.2–21.45) | 849.82(659.42–1102.76) | 13.1(10.18–17.09) | 79.25 | -1.07(-1.29–-0.85) |
| Indonesia | 104.04(64.74–153.89) | 15.86(10.02–23.65) | 170.54(113.95–268.18) | 12.81(8.63–20.42) | 63.91 | -0.7(-0.82–-0.58) |
| Iran  (Islamic Republic of) | 8.35(5.81–9.77) | 5.36(3.78–6.38) | 16.8(12.22–19.33) | 3.99(2.87–4.57) | 101.11 | -1.28(-1.48–-1.09) |
| Iraq | 2.16(1.55–3.03) | 4.65(3.39–6.46) | 6.89(4.8–9.69) | 4.61(3.25–6.33) | 219.8 | -0.03(-0.2–0.14) |
| Ireland | 2.1(1.85–2.34) | 11.25(9.72–12.54) | 3.04(1.99–4.09) | 9.64(6.23–13.04) | 44.89 | -0.11(-0.3–0.08) |
| Israel | 1.58(1.41–1.86) | 6.39(5.69–7.51) | 3.45(2.44–4.54) | 6.65(4.63–8.83) | 118.65 | -0.03(-0.16–0.1) |
| Italy | 20.28(19.03–24.84) | 5.06(4.73–6.21) | 39.48(23.08–51.36) | 7.57(4.48–9.94) | 94.69 | 2.02(1.7–2.34) |
| Jamaica | 2.88(2.52–3.15) | 32.08(27.97–35.05) | 5.21(3.84–6.87) | 34.14(25.15–45.04) | 80.9 | 0.21(-0.1–0.53) |
| Japan | 90.48(85.15–100.8) | 10.48(9.86–11.62) | 111.05(81.01–140.16) | 11.17(7.66–14.29) | 22.74 | 0.79(0.59–0.99) |
| Jordan | 0.52(0.38–0.67) | 6.04(4.56–7.8) | 1.6(1.11–2.15) | 4.03(2.88–5.36) | 209.09 | -1.66(-1.86–-1.46) |
| Kazakhstan | 16.78(15.23–18.68) | 21.28(19.4–23.58) | 19.39(16.08–23.49) | 18.48(15.33–22.34) | 15.59 | 0.06(-0.2–0.33) |
| Kenya | 9.79(6.44–16.33) | 18.54(12.33–30.34) | 28.14(18.44–45.47) | 18.3(12.09–29.19) | 187.47 | -0.17(-0.33–-0.01) |
| Kiribati | 0.32(0.24–0.41) | 127.98(97.33–164.28) | 0.51(0.36–0.67) | 108.8(78.79–140.73) | 60.72 | -0.61(-0.69–-0.53) |
| Kuwait | 0.25(0.2–0.3) | 6.32(5.08–7.49) | 0.64(0.47–0.93) | 3.62(2.69–5.1) | 155.63 | -1.59(-1.84–-1.34) |
| Kyrgyzstan | 4.15(3.55–4.57) | 23.24(20.05–25.54) | 5.5(4.38–6.6) | 18.27(14.68–21.87) | 32.55 | -0.72(-0.98–-0.47) |
| Lao People's Democratic Republic | 3.37(1.72–5.03) | 25.47(13.6–38.45) | 4.51(2.75–6.71) | 15.69(9.64–23.64) | 33.68 | -1.93(-2.03–-1.83) |
| Latvia | 2.68(2.33–2.92) | 13.89(12.24–15.18) | 1.45(1.06–2.01) | 8.68(6.19–12.16) | -45.66 | -1.68(-1.96–-1.4) |
| Lebanon | 0.94(0.66–1.22) | 7.32(5.24–9.43) | 1.77(1.18–2.49) | 6.08(4.06–8.52) | 87.22 | -0.73(-0.85–-0.61) |
| Lesotho | 1.67(1.09–2.47) | 28.52(18.78–42.2) | 4.35(2.15–7.49) | 52.77(26.49–90.4) | 160.71 | 3.43(2.9–3.95) |
| Liberia | 2.04(1.54–2.75) | 34.53(26.42–46.17) | 4.37(2.89–6.28) | 30.99(20.85–43.31) | 113.9 | -0.39(-0.5–-0.27) |
| Libya | 0.94(0.64–1.23) | 9(6.16–11.68) | 2.77(1.73–3.79) | 8.29(5.44–11.2) | 193.08 | -0.1(-0.38–0.19) |
| Lithuania | 4.59(3.89–4.98) | 19.07(16.44–20.81) | 2.97(2.31–3.69) | 12.43(9.48–15.81) | -35.15 | -1.36(-1.59–-1.13) |
| Luxembourg | 0.26(0.23–0.29) | 10.26(8.9–11.56) | 0.23(0.18–0.3) | 5.32(4.21–6.97) | -13.25 | -2.25(-2.33–-2.17) |
| Madagascar | 13.63(9.49–18.16) | 40.04(27.78–52.83) | 27.5(17.53–40.53) | 33.54(21.59–48.54) | 101.82 | -0.7(-0.77–-0.64) |
| Malawi | 11.78(8.48–15.76) | 44.52(32.34–59.2) | 20.89(13.33–31.46) | 39.8(25.91–57.57) | 77.35 | -0.46(-0.68–-0.25) |
| Malaysia | 12.4(8.31–14.35) | 21.85(14.51–25.15) | 26.02(18.49–34.46) | 17.92(12.6–23.65) | 109.91 | -1.09(-1.37–-0.81) |
| Maldives | 0.12(0.05–0.17) | 22.18(9.36–31.64) | 0.15(0.12–0.2) | 8.78(6.85–11.47) | 29.72 | -3.68(-4–-3.35) |
| Mali | 9.12(6.89–11.31) | 35.28(26.85–43.72) | 15.38(10.63–21.51) | 28.07(19.56–38.89) | 68.63 | -1.04(-1.14–-0.93) |
| Malta | 0.17(0.15–0.19) | 7.46(6.46–8.49) | 0.17(0.13–0.21) | 4.94(3.92–6.28) | -1.33 | -1.02(-1.25–-0.79) |
| Marshall Islands | 0.04(0.03–0.06) | 36.76(24.75–56.62) | 0.09(0.05–0.14) | 37.9(21.94–60.33) | 123.79 | 0.03(-0.1–0.16) |
| Mauritania | 2.39(1.69–3.16) | 40.08(28.38–53.02) | 3.51(2.39–4.98) | 28.01(19.42–38.91) | 46.5 | -1.04(-1.14–-0.94) |
| Mauritius | 0.81(0.71–0.9) | 18.22(16.17–20.28) | 1.08(0.84–1.42) | 12.09(9.34–15.7) | 33.92 | -2.07(-2.29–-1.85) |
| Mexico | 95.84(81.78–100.62) | 36.09(29.87–37.98) | 121.95(96.56–165.27) | 18.34(14.55–24.83) | 27.25 | -2.77(-2.97–-2.56) |
| Micronesia  (Federated States of) | 0.11(0.07–0.17) | 39.56(25.6–62.06) | 0.16(0.09–0.26) | 36.21(20.8–58.83) | 41.07 | -0.29(-0.42–-0.17) |
| Monaco | 0.03(0.02–0.03) | 11.05(7.83–14.09) | 0.02(0.02–0.03) | 8.47(6.02–11.93) | -9 | -0.78(-0.96–-0.6) |
| Mongolia | 1.81(1.41–2.42) | 29.05(22.7–38.61) | 3.47(2.44–5.08) | 20.97(15.17–29.49) | 91.54 | -1.55(-1.73–-1.37) |
| Montenegro | 0.45(0.37–0.58) | 13.48(10.9–17.33) | 0.57(0.45–0.72) | 13.58(10.71–17.24) | 25.25 | -0.02(-0.37–0.32) |
| Morocco | 11.28(7.53–13.93) | 13.89(9.31–17.07) | 25.42(15.92–35.85) | 14.03(9.16–19.52) | 125.39 | 0.05(-0.03–0.13) |
| Mozambique | 15.84(10.21–22.9) | 39.09(25.49–55.66) | 34.66(20.63–52.14) | 43.21(26.52–63.97) | 118.74 | 0.56(0.29–0.82) |
| Myanmar | 35.97(19.48–56.38) | 24.27(13.6–38.86) | 41.42(27.94–66.64) | 14.25(9.64–23.27) | 15.16 | -2.16(-2.37–-1.96) |
| Namibia | 0.88(0.58–1.27) | 20.6(13.68–29.94) | 2.5(1.6–3.75) | 26.93(17.67–39.93) | 184.08 | 1.17(1.03–1.31) |
| Nauru | 0.01(0.01–0.02) | 43.03(28.31–64.97) | 0.01(0.01–0.02) | 39.5(23.23–59.83) | 19.22 | -0.3(-0.44–-0.17) |
| Nepal | 13.48(6.26–18.49) | 22.23(10.42–30.33) | 18.91(11.63–27.57) | 13.91(8.66–20.11) | 40.2 | -1.69(-2.12–-1.26) |
| Netherlands | 7.85(6.99–8.53) | 8.34(7.41–9.11) | 8.42(6.31–10.78) | 6.97(5.11–9.13) | 7.37 | -0.71(-0.82–-0.6) |
| New Zealand | 2.76(1.84–3.04) | 14.52(9.5–16.06) | 2.02(1.52–2.64) | 6.36(4.75–8.52) | -26.93 | -2.59(-3.04–-2.14) |
| Nicaragua | 3.88(3.13–4.46) | 36.36(28.9–41.26) | 8.29(6.4–11.2) | 29.76(23.27–40.25) | 113.53 | -0.8(-0.97–-0.63) |
| Niger | 6.51(4.73–9.05) | 35.16(25.74–48.01) | 17.63(12.05–24.81) | 33.98(23.54–46.95) | 170.62 | -0.26(-0.34–-0.18) |
| Nigeria | 47.57(30.15–67.87) | 20.13(12.91–28.12) | 112.56(73.01–166.05) | 18.23(12.14–26.47) | 136.61 | -0.19(-0.27–-0.11) |
| Niue | 0(0–0) | 28.7(20.47–43.16) | 0(0–0) | 24.91(15.7–37.74) | -19.23 | -0.66(-0.76–-0.55) |
| North Macedonia | 1.52(1.32–1.98) | 14.58(12.71–19.21) | 2.13(1.52–2.86) | 14.51(10.49–19.37) | 40.56 | -0.6(-1.06–-0.15) |
| Northern Mariana Islands | 0.07(0.05–0.09) | 46.19(34.09–61.58) | 0.1(0.07–0.13) | 36.17(26.58–47.24) | 48.73 | -0.89(-1.03–-0.75) |
| Norway | 3.39(3.13–3.65) | 12.53(11.33–13.52) | 2.9(2.24–3.71) | 8.04(6.16–10.38) | -14.39 | -1.58(-1.64–-1.51) |
| Oman | 0.29(0.2–0.4) | 7.73(5.28–10.54) | 0.63(0.45–0.82) | 5.94(4.46–7.43) | 116.73 | -0.65(-0.98–-0.31) |
| Pakistan | 21.96(17.59–27.74) | 6.97(5.63–8.84) | 56.99(40.25–81.09) | 7.7(5.51–10.8) | 159.54 | 0.09(-0.14–0.32) |
| Palau | 0.05(0.03–0.06) | 77.73(55.03–107.52) | 0.07(0.05–0.1) | 66.58(47.58–89.82) | 60.68 | -0.54(-0.64–-0.43) |
| Palestine | 0.31(0.2–0.42) | 5.95(3.84–8) | 0.67(0.44–0.83) | 4.66(2.97–5.72) | 113.96 | -0.88(-1.21–-0.55) |
| Panama | 3.26(2.47–3.59) | 36.87(28.26–40.35) | 4.76(3.45–6.36) | 22.52(16.33–30.15) | 45.99 | -1.96(-2.24–-1.68) |
| Papua New Guinea | 2.64(1.56–4.16) | 22.17(13.33–36.04) | 7.77(4.39–11.58) | 23.52(13.91–36.42) | 194.06 | 0.32(0.26–0.39) |
| Paraguay | 4.48(3.47–5.3) | 33.09(25.65–39.06) | 10.9(7.52–14.88) | 34.26(23.77–46.57) | 143.35 | -0.22(-0.46–0.02) |
| Peru | 22.19(18.33–27.64) | 31.09(25.82–38.41) | 47.77(32.69–65.46) | 27.64(18.91–37.77) | 115.29 | -0.62(-0.84–-0.4) |
| Philippines | 33.58(24.73–39.84) | 16.44(12.72–19.81) | 67.76(47.23–91.41) | 13.9(9.78–18.89) | 101.77 | -0.59(-0.78–-0.4) |
| Poland | 41.84(38.29–43.83) | 18(16.5–18.88) | 32.52(24.47–41.95) | 10.53(7.88–13.77) | -22.28 | -2.1(-2.28–-1.91) |
| Portugal | 10.19(9.17–11.15) | 15.79(14.05–17.36) | 9.36(6.97–12.26) | 10.37(7.61–13.77) | -8.09 | -1.76(-2.02–-1.5) |
| Puerto Rico | 2.15(1.93–2.38) | 11.2(10.03–12.43) | 2.76(2.05–3.65) | 10.86(7.82–14.73) | 28.3 | -0.01(-0.16–0.13) |
| Qatar | 0.06(0.05–0.09) | 9.47(7.14–12.92) | 0.33(0.23–0.47) | 8.34(6.26–11.04) | 432.19 | -0.08(-0.36–0.19) |
| Republic of Korea | 31.1(25.88–36.41) | 15.02(12.84–18.23) | 35.97(27.87–46.61) | 9.08(6.98–11.85) | 15.66 | -2.34(-2.59–-2.09) |
| Republic of Moldova | 5.28(4.44–5.75) | 20.71(17.37–22.58) | 3.99(3.15–4.93) | 14.77(11.43–18.42) | -24.46 | -0.51(-0.79–-0.22) |
| Romania | 39.66(36.73–42.43) | 29.28(27.1–31.47) | 39.32(27.36–49.65) | 27.36(19.1–34.98) | -0.85 | -0.53(-0.82–-0.25) |
| Russian Federation | 124.38(117.3–137.59) | 12.1(11.38–13.49) | 165.17(123.3–210.65) | 15.66(11.27–20.22) | 32.79 | 1.02(0.79–1.26) |
| Rwanda | 10.93(7.33–15.07) | 54.02(37.19–73.18) | 14.21(9.22–21.99) | 32.39(21.4–48.8) | 30.05 | -2.53(-2.86–-2.2) |
| Saint Kitts and Nevis | 0.11(0.1–0.13) | 62.98(53.94–72.76) | 0.11(0.07–0.14) | 29.07(18.42–40.35) | -7.24 | -2.66(-2.83–-2.49) |
| Saint Lucia | 0.21(0.18–0.23) | 42.02(37.16–46.67) | 0.31(0.25–0.38) | 28.52(22.96–34.91) | 49.4 | -1.64(-1.86–-1.42) |
| Saint Vincent and the Grenadines | 0.2(0.17–0.22) | 50.46(44.23–56.94) | 0.26(0.21–0.31) | 41.01(33.53–49.57) | 31.61 | -1.04(-1.21–-0.88) |
| Samoa | 0.13(0.08–0.18) | 26.06(17.18–35.45) | 0.21(0.13–0.31) | 25.68(15.39–37.17) | 62.34 | -0.12(-0.18–-0.05) |
| San Marino | 0.01(0.01–0.01) | 5.51(4.34–7.34) | 0.02(0.01–0.02) | 6.75(4.68–9.24) | 98.95 | 1.17(0.96–1.39) |
| Sao Tome and Principe | 0.15(0.1–0.18) | 40.54(28.31–50.67) | 0.31(0.21–0.43) | 44.01(30.62–59.89) | 113.64 | -0.05(-0.31–0.21) |
| Saudi Arabia | 1.15(0.78–1.95) | 3.65(2.57–6.11) | 6.38(4.4–9.2) | 4.95(3.65–6.79) | 453.6 | 1.59(1.31–1.87) |
| Senegal | 6.34(4.63–8.53) | 31.6(23.39–41.9) | 14.09(9.93–18.94) | 29.88(21.46–39.78) | 122.07 | 0.01(-0.19–0.22) |
| Serbia | 14.46(11.61–16.95) | 25.62(20.59–30.07) | 13.7(9.98–17.98) | 22.27(16.09–29.46) | -5.22 | -0.86(-1.1–-0.63) |
| Seychelles | 0.12(0.1–0.15) | 40.81(34.17–49.11) | 0.2(0.15–0.26) | 33.51(26.38–44.12) | 61.98 | -0.55(-0.62–-0.48) |
| Sierra Leone | 3.03(2.13–4.19) | 27.64(19.64–37.83) | 8.04(5.15–11.46) | 34(22.22–48.48) | 164.77 | 1.09(0.89–1.29) |
| Singapore | 2.78(2.37–3.08) | 19.08(16.31–21.03) | 3.06(2.36–4) | 7.7(5.91–10.05) | 9.98 | -3.4(-3.61–-3.18) |
| Slovakia | 5.75(4.83–6.57) | 18.77(15.76–21.46) | 6.65(4.28–8.81) | 16.72(10.72–22.36) | 15.59 | -0.17(-0.36–0.02) |
| Slovenia | 2.11(1.54–2.83) | 16.88(12.19–22.76) | 1.58(1.14–2.21) | 10.03(7.11–14.34) | -24.83 | -2.06(-2.24–-1.89) |
| Solomon Islands | 0.49(0.2–0.82) | 55.51(25.27–93.57) | 1.33(0.53–2.07) | 57(25.78–86.4) | 170.77 | 0.15(0.09–0.21) |
| Somalia | 9.47(5.46–14.38) | 47.7(27.23–70.63) | 21.31(11.68–33.2) | 42.18(23.88–65.21) | 125.09 | -0.25(-0.31–-0.18) |
| South Africa | 45.47(34.59–56.82) | 31.75(23.92–40.01) | 82.46(66.14–101.63) | 29.19(23.67–35.87) | 81.35 | -0.03(-0.28–0.22) |
| South Sudan | 4.23(2.62–6.19) | 31.87(19.88–45.37) | 6.59(3.85–11.04) | 25.59(15.57–41.43) | 55.94 | -0.72(-0.84–-0.6) |
| Spain | 25.97(22.14–28.14) | 10.66(8.96–11.6) | 32.63(22.17–42.93) | 8.7(5.98–11.52) | 25.62 | -0.61(-0.75–-0.48) |
| Sri Lanka | 5.11(4.27–6.92) | 7.69(6.48–10.6) | 10.77(7.29–14.99) | 7.84(5.34–10.94) | 110.79 | 0.18(-0.05–0.41) |
| Sudan | 3.64(1.63–5.06) | 6.63(2.97–9.08) | 6.63(3.73–9.98) | 5.49(3.2–8.02) | 82.33 | -0.66(-0.72–-0.59) |
| Suriname | 0.52(0.41–0.6) | 34.36(27.68–39.63) | 1.05(0.8–1.34) | 32.61(24.95–41.91) | 102.07 | -0.44(-0.68–-0.21) |
| Sweden | 5.7(5.21–6.57) | 9.8(8.9–11.18) | 4.96(3.87–6.25) | 6.93(5.34–8.8) | -13 | -1.11(-1.21–-1) |
| Switzerland | 4.95(3.73–5.45) | 10.4(8.07–11.57) | 3.93(2.98–5.17) | 5.73(4.26–7.62) | -20.59 | -2.21(-2.31–-2.1) |
| Syrian Arab Republic | 1.26(0.87–1.7) | 3.87(2.74–5.11) | 2.19(1.55–3.13) | 3.25(2.33–4.61) | 74.21 | -0.74(-0.94–-0.55) |
| Taiwan  (Province of China) | 24.7(21.49–26.29) | 28.82(25.38–30.63) | 24.72(18.52–34.73) | 13.09(9.72–18.42) | 0.06 | -3.63(-3.96–-3.3) |
| Tajikistan | 2.15(1.52–2.5) | 13.01(9.13–15.05) | 2.63(1.95–4.32) | 7.65(5.82–11.86) | 22.36 | -1.86(-2.35–-1.37) |
| Thailand | 65.05(47.05–77.66) | 27.56(20.26–32.65) | 83.35(58.35–122.53) | 16.27(11.26–24.12) | 28.15 | -2.46(-2.8–-2.12) |
| Timor-Leste | 0.38(0.21–0.55) | 18.49(10.81–27.16) | 0.69(0.4–1.05) | 15.52(9.4–23.67) | 79.98 | -0.78(-1.09–-0.46) |
| Togo | 3.32(2.55–4.44) | 35.77(28.09–47.67) | 8.4(5.81–11.72) | 30.94(21.92–42.44) | 152.9 | -0.41(-0.5–-0.31) |
| Tokelau | 0(0–0) | 40.45(27.1–62.56) | 0(0–0) | 33.62(22.27–50.14) | -17.54 | -0.67(-0.73–-0.61) |
| Tonga | 0.12(0.09–0.15) | 37.28(28.73–46.48) | 0.14(0.1–0.19) | 31.45(22.53–43.86) | 15.41 | -0.72(-0.87–-0.57) |
| Trinidad and Tobago | 1.43(1.3–1.62) | 30.23(27.49–33.7) | 2.01(1.45–2.71) | 22.28(16.03–30.26) | 40.43 | -1.41(-1.58–-1.23) |
| Tunisia | 1.85(1.33–2.31) | 6.43(4.7–7.99) | 3.89(2.55–5.44) | 5.72(3.79–7.98) | 110.08 | -0.46(-0.51–-0.42) |
| Turkey | 14.58(9.14–18.41) | 6.91(4.39–8.65) | 22.02(15.29–28.28) | 4.67(3.23–5.99) | 50.97 | -1.34(-1.66–-1.01) |
| Turkmenistan | 1.84(1.66–2.03) | 14.79(13.27–16.35) | 3.9(2.76–5.21) | 15.59(11.08–20.66) | 112.27 | 1.03(0.62–1.44) |
| Tuvalu | 0.02(0.01–0.03) | 40.83(25.84–61.9) | 0.02(0.01–0.03) | 32.26(21.54–48.65) | -1.81 | -0.81(-0.89–-0.74) |
| Uganda | 13.85(9.48–18.92) | 33.39(23.39–44.86) | 39.97(27.91–53.94) | 37.92(26.74–49.75) | 188.58 | 0.12(-0.12–0.35) |
| Ukraine | 77.37(52.13–84.53) | 19.66(13.26–21.51) | 43.55(32.39–58.79) | 11.91(8.76–16.42) | -43.72 | -2.32(-2.53–-2.11) |
| United Arab Emirates | 0.32(0.22–0.44) | 16.83(11.34–23.71) | 1.9(1.3–2.83) | 10.82(7.66–14.68) | 501.27 | -1.4(-2.03–-0.77) |
| United Kingdom | 51.94(43.75–53.74) | 13.97(10.98–14.48) | 36.69(28.11–48.14) | 8.19(6.19–10.71) | -29.36 | -1.71(-1.99–-1.44) |
| United Republic of Tanzania | 27.49(18.59–37.37) | 39.53(26.76–53.08) | 58.95(38.74–85.58) | 35.17(23.77–49.46) | 114.48 | -0.34(-0.49–-0.19) |
| United States of America | 156.56(132.28–163.61) | 10.28(8.59–10.73) | 191.12(148.85–235.73) | 8.67(6.7–10.73) | 22.07 | -0.68(-0.82–-0.54) |
| United States Virgin Islands | 0.11(0.09–0.14) | 21.53(16.77–26.18) | 0.13(0.1–0.16) | 15.46(11.43–20.33) | 11.95 | -1.17(-1.26–-1.09) |
| Uruguay | 4.2(3.81–4.66) | 23.84(21.49–26.51) | 4.82(3.57–6.31) | 21.81(15.92–28.93) | 14.84 | -0.47(-0.63–-0.31) |
| Uzbekistan | 10.71(9.58–12) | 15.26(13.69–16.92) | 27.04(21.01–33.47) | 17.26(13.49–21.22) | 152.38 | 0.18(-0.05–0.4) |
| Vanuatu | 0.11(0.06–0.16) | 27.06(15.08–41.78) | 0.29(0.15–0.47) | 28.7(15.49–44.39) | 175.68 | -0.23(-0.46–0) |
| Venezuela | 22.21(20.49–24.27) | 34.11(31.52–37.53) | 54.28(38.63–74.63) | 34.36(24.47–47.24) | 144.43 | -0.31(-0.52–-0.11) |
| Viet Nam | 42.5(31.47–55.48) | 17.58(13.09–23.22) | 99.66(69.56–131.58) | 17.77(12.52–23.37) | 134.48 | 0.03(-0.09–0.15) |
| Yemen | 2.14(0.96–3.32) | 6.92(3.27–10.42) | 5.73(3.41–8.71) | 6.39(3.98–9.4) | 167.8 | -0.29(-0.36–-0.22) |
| Zambia | 10.23(7.08–13.54) | 52.18(36.49–67.98) | 21.89(14.36–30.98) | 43.37(28.91–60.93) | 114.01 | -1.03(-1.2–-0.87) |
| Zimbabwe | 11.57(8.11–14.96) | 45.33(31.58–58.26) | 24.49(16.32–34.11) | 48.95(33.04–67.77) | 111.68 | 0.85(0.52–1.18) |

EAPC: estimated annual percentage change; ASR, age-standardized rate; CI, confidence interval; UI: uncertainty interval.
