# Supplementary material for: Trends of cervical cancer at global, regional, and national level: data from the Global Burden of Disease study 2019
Source: BMC Public Health. 2021 May 12;21:894. doi: 10.1186/s12889-021-10907-5 (PMC8114503; doi:10.1186/s12889-021-10907-5)
Supplement: Supplementary file 7 — Additional file 7: Supplementary Table 3. The number and age-standardized rate of death due to cervical cancer in global, sexes, SDI areas and geographic regions in 1990 and 2019, and percentage change of absolute number and the EAPCs from 1990 to 2019. [file 12889_2021_10907_MOESM7_ESM.doc]

**Supplementary Table 3.** The number and age-standardized rate of death due to cervical cancer in global, sexes, SDI areas and geographic regions in 1990 and 2019, and percentage change of absolute number and the EAPCs from 1990 to 2019

|  | 1990 | | 2019 | | 1990–2019 | |
| --- | --- | --- | --- | --- | --- | --- |
| **Characteristics** | Number  ×10^3^ (95% UI) | ASR/100,000)  (95% UI) | Number  ×10^3^ (95% UI) | ASR/100,000)  (95% UI) | Change in  number (%) | EAPCs  (95%CI) |
| **Overall** | 184.53  (164.84–218.94) | 8.48  (7.59–10.07) | 280.48  (238.86–313.93) | 6.51  (5.55–7.29) | 52.00 | -0.93  (-0.98–-0.88) |
| **SDI** |  |  |  |  |  |  |
| Low | 26.08  (20.23–32.11) | 19.18  (15–23.66) | 45.54  (35.8–56.26) | 15.05  (11.92–18.46) | 74.62 | -0.90  (-0.94–-0.87) |
| Low-middle | 39.21  (32.46–50.05) | 11.71  (9.73–15.05) | 66.68  (57.27–81.24) | 8.85  (7.62–10.83) | 70.06 | -1.04  (-1.12–-0.96) |
| Middle | 52.53  (46.63–65.12) | 9.32  (8.31–11.54) | 90.10  (71.33–103.20) | 6.78  (5.4–7.76) | 71.53 | -1.03  (-1.09–-0.97) |
| High-middle | 41.35  (38.69–48.4) | 6.95  (6.5–8.13) | 51.77  (41.66–57.87) | 4.89  (3.92–5.47) | 25.19 | -1.25  (-1.31–-1.19) |
| High | 25.22  (23.28–26.19) | 4.56  (4.22–4.71) | 26.17  (22.82–28.15) | 2.90  (2.60–3.10) | 3.77 | -1.57  (-1.68–-1.46) |
| **Regions** |  |  |  |  |  |  |
| East Asia | 28.40  (22.32–46.14) | 6.05  (4.77–9.76) | 55.96  (33.19–71.36) | 5.18  (3.09–6.59) | 97.01 | -0.05  (-0.28–0.18) |
| South Asia | 33.34  (26.22–39.95) | 10.49  (8.29–12.62) | 53.30  (42.87–69.95) | 7.01  (5.66–9.21) | 59.89 | -1.60  (-1.77–-1.42) |
| Southeast Asia | 16.71  (12.57–21.47) | 11.05  (8.36–14.54) | 25.13  (20.52–34.98) | 7.36  (6.03–10.33) | 50.34 | -1.52  (-1.61–-1.44) |
| Central Asia | 2.72  (2.48–2.89) | 9.81  (8.95–10.43) | 3.42  (3.00–3.93) | 7.58  (6.68–8.7) | 25.87 | -0.75  (-0.88–-0.62) |
| High-income  Asia Pacific | 4.64(  4.35–5.32) | 4.20  (3.93–4.81) | 5.60  (4.58–6.22) | 2.70  (2.22–2.96) | 20.71 | -1.52  (-1.59–-1.44) |
| Oceania | 0.30  (0.22–0.42) | 18.16  (13.38–25.22) | 0.67  (0.45–0.91) | 16.41  (11.5–22.19) | 119.36 | -0.18  (-0.27–-0.08) |
| Australasia | 0.45  (0.39–0.48) | 3.73  (3.16–3.92) | 0.52  (0.45–0.58) | 2.17  (1.88–2.40) | 15.35 | -1.57  (-1.95–-1.19) |
| Eastern Europe | 12.94  (10.95–13.86) | 7.62  (6.50–8.19) | 10.04  (8.47–11.91) | 5.54  (4.62–6.61) | -22.42 | -1.38  (-1.54–-1.21) |
| Western Europe | 13.13  (12.04–13.61) | 4.36  (3.99–4.51) | 11.75  (10.27–12.69) | 2.65  (2.38–2.85) | -10.49 | -1.65  (-1.75–-1.54) |
| Central Europe | 8.00  (7.60–8.53) | 10.14  (9.63–10.79) | 6.88  (5.82–7.99) | 6.65  (5.59–7.75) | -13.94 | -1.57  (-1.68–-1.47) |
| High-income North America | 6.74  (5.97–7.04) | 3.71  (3.25–3.86) | 8.80  (7.47–9.34) | 2.99  (2.55–3.15) | 30.51 | -0.69  (-0.83–-0.55) |
| Andean Latin America | 2.33  (1.96–2.76) | 20.39  (17.22–24.05) | 4.28  (3.32–5.38) | 14.37  (11.18–18.04) | 83.52 | -1.33  (-1.44–-1.22) |
| Central Latin America | 9.59  (8.70–10.01) | 20.35  (18.19–21.28) | 13.83  (11.53–16.8) | 10.65  (8.91–12.92) | 44.28 | -2.61  (-2.76–-2.46) |
| Caribbean | 2.23  (1.75–2.55) | 15.83  (12.55–18.04) | 3.47  (2.72–4.26) | 12.95  (10.11–15.96) | 55.78 | -0.65  (-0.72–-0.58) |
| Tropical Latin America | 7.68  (7.21–8.88) | 14.72  (13.7–17.12) | 11.58  (10.71–13.66) | 8.69  (8.04–10.23) | 50.69 | -2.01  (-2.11–-1.92) |
| Southern Latin America | 3.07  (2.89–3.31) | 12.34  (11.62–13.3) | 4.18  (3.55–4.60) | 9.64  (8.17–10.56) | 36.17 | -1.01  (-1.12–-0.89) |
| Eastern Sub-  Saharan Africa | 11.94  (9.08–14.92) | 26.51  (20.07–33.45) | 21.11  (15.48–27.86) | 21.13  (15.15–27.62) | 76.86 | -0.90  (-0.96–-0.84) |
| Southern Sub-  Saharan Africa | 3.24  (2.48–4.07) | 19.17  (14.63–24.27) | 6.56  (5.39–7.75) | 19.34  (15.82–22.77) | 102.54 | 0.46  (0.19–0.72) |
| Western Sub-  Saharan Africa | 9.38  (7.56–12.31) | 19.74  (15.94–25.76) | 19.09  (15.04–24.01) | 16.83  (13.38–21) | 103.53 | -0.48  (-0.53–-0.43) |
| North Africa and Middle East | 3.97  (2.82–4.53) | 4.37  (3.10–4.99) | 7.00  (5.44–8.31) | 3.15  (2.47–3.69) | 76.40 | -1.11  (-1.21–-1.01) |
| Central Sub-  Saharan Africa | 3.72  (2.61–4.84) | 26.27  (18.78–34.25) | 7.30  (4.91–10.06) | 21.67  (14.49–30.24) | 96.10 | -0.67  (-0.78–-0.55) |

EAPC: estimated annual percentage change; ASR, age-standardized rate; CI, confidence interval; UI: uncertainty interval; SDI: socio-demographic index. Percentage change in absolute number was calculated based on the crew data.
