# Supplementary material for: Trends of cervical cancer at global, regional, and national level: data from the Global Burden of Disease study 2019
Source: BMC Public Health. 2021 May 12;21:894. doi: 10.1186/s12889-021-10907-5 (PMC8114503; doi:10.1186/s12889-021-10907-5)
Supplement: Supplementary file 8 — Additional file 8: Supplementary Table 4. the number and age-standardized rate of death caused by cervical cancer at national level and both sexes in 1990 and 2019, and the percentage changes in number and the EAPCs from 1990 to 2019. [file 12889_2021_10907_MOESM8_ESM.doc]

**Supplementary Table 4**. the number and age-standardized rate of death caused by cervical cancer at national level and both sexes in 1990 and 2019, and the percentage changes in number and the EAPCs from 1990 to 2019

|  | **1990** | | **2019** | | **1990–2019** | |
| --- | --- | --- | --- | --- | --- | --- |
| **Characteristics** | Number  ×10^3^ (95% UI) | ASR/100,000)  (95% UI) | Number  ×10^3^ (95% UI) | ASR/100,000)  (95% UI) | Changes in number (%) | EAPC  (95%CI) |
| Afghanistan | 3.32(1.11–5.08) | 8.96(3.18–13.47) | 6.09(2.39–9.39) | 7.59(3.4–11.25) | 83.19 | -0.71(-0.89–-0.52) |
| Albania | 0.45(0.39–0.64) | 3.85(3.38–5.63) | 0.64(0.45–0.88) | 3.13(2.22–4.32) | 43.1 | -0.45(-0.64–-0.27) |
| Algeria | 4.94(3.44–6.41) | 7.61(5.56–9.73) | 7.87(5.62–10.62) | 4.52(3.3–5.95) | 59.1 | -1.73(-1.85–-1.6) |
| American Samoa | 0.01(0.01–0.02) | 9.75(7.97–13.11) | 0.02(0.02–0.03) | 9.59(7.22–12.23) | 105.42 | 0.04(-0.16–0.23) |
| Andorra | 0.01(0.01–0.02) | 5.22(3.75–7.35) | 0.03(0.02–0.04) | 4.1(2.9–5.64) | 103.97 | -0.87(-1.09–-0.64) |
| Angola | 6.36(4.19–9.21) | 26.12(17.31–37.21) | 14.61(9.28–21.48) | 19.85(13.12–29.7) | 129.76 | -1.11(-1.25–-0.96) |
| Antigua and Barbuda | 0.03(0.03–0.04) | 11.95(10.43–13.75) | 0.06(0.05–0.07) | 10.81(8.89–12.98) | 69 | -0.6(-0.76–-0.43) |
| Argentina | 18.53(17.35–21.82) | 10.85(10.15–12.7) | 30.35(24.09–33.54) | 10.85(8.52–11.96) | 63.75 | -0.11(-0.28–0.07) |
| Armenia | 1.52(1.4–1.68) | 9.55(8.77–10.55) | 1.58(1.27–1.91) | 7.09(5.72–8.59) | 3.81 | -1.03(-1.25–-0.81) |
| Australia | 3.45(3.13–3.65) | 3.37(3.03–3.57) | 4.36(3.64–4.88) | 2.14(1.8–2.37) | 26.52 | -1.27(-1.64–-0.9) |
| Austria | 4.28(3.57–4.59) | 6.56(5.43–7.01) | 2.39(2.12–3.02) | 2.66(2.37–3.36) | -44.13 | -3.26(-3.49–-3.04) |
| Azerbaijan | 2.25(1.95–2.82) | 7.44(6.43–9.42) | 3.34(2.48–4.77) | 6.04(4.56–8.72) | 48.08 | -0.79(-0.94–-0.65) |
| Bahamas | 0.14(0.12–0.16) | 14.31(12.51–16.19) | 0.24(0.19–0.31) | 10.85(8.48–13.77) | 75.14 | -1.08(-1.19–-0.98) |
| Bahrain | 0.04(0.04–0.06) | 4.98(4.06–6.6) | 0.11(0.09–0.16) | 2.96(2.3–3.99) | 165.97 | -1.89(-2.09–-1.69) |
| Bangladesh | 32.44(14.69–42.35) | 12.99(5.87–16.83) | 38.64(21.5–58.41) | 5.67(3.14–8.52) | 19.13 | -2.69(-2.88–-2.49) |
| Barbados | 0.27(0.24–0.29) | 17.22(15.41–18.83) | 0.33(0.27–0.4) | 13.11(10.57–16.04) | 22.91 | -0.82(-0.9–-0.74) |
| Belarus | 6.36(5.9–6.97) | 8.35(7.77–9.12) | 5.07(3.82–6.93) | 5.9(4.41–8.08) | -20.2 | -1.57(-1.75–-1.39) |
| Belgium | 3.69(3.02–3.98) | 4.52(3.79–4.84) | 3.06(2.57–3.42) | 2.69(2.31–2.99) | -17.06 | -1.82(-2–-1.64) |
| Belize | 0.1(0.09–0.11) | 20.28(17.89–22.83) | 0.28(0.23–0.33) | 17.66(14.59–20.77) | 182.36 | -0.64(-0.98–-0.3) |
| Benin | 2.59(2.06–3.39) | 22.62(18.1–29.48) | 5.94(4.26–8.28) | 20.12(14.87–27.41) | 129.2 | -0.3(-0.35–-0.25) |
| Bermuda | 0.03(0.02–0.03) | 7.75(6.72–8.86) | 0.02(0.02–0.03) | 3.25(2.59–4.1) | -20.28 | -3.56(-3.81–-3.3) |
| Bhutan | 0.2(0.09–0.28) | 13.55(6.25–18.92) | 0.21(0.13–0.32) | 7.24(4.35–11.01) | 6.09 | -2.38(-2.62–-2.14) |
| Bolivia | 6.16(4–7.77) | 33.21(21.89–41.64) | 11.38(8.11–15.45) | 24.14(17.53–32.4) | 84.72 | -1.25(-1.36–-1.14) |
| Bosnia and Herzegovina | 1.51(1.32–1.8) | 6.35(5.62–7.64) | 1.63(1.16–2.09) | 5.61(3.89–7.24) | 8.06 | -0.57(-0.74–-0.39) |
| Botswana | 0.84(0.54–1.26) | 24.57(16.15–35.97) | 2.31(1.37–3.59) | 26.7(16.4–40.82) | 175.57 | 0.23(-0.03–0.5) |
| Brazil | 74.48(69.86–85.83) | 14.63(13.63–16.94) | 110.75(102.42–131.72) | 8.51(7.87–10.1) | 48.68 | -2.06(-2.15–-1.97) |
| Brunei Darussalam | 0.11(0.08–0.13) | 17.51(13.31–21.37) | 0.18(0.15–0.24) | 10.46(8.59–13.36) | 73.77 | -1.79(-1.93–-1.66) |
| Bulgaria | 4.53(4.1–5.13) | 7.47(6.73–8.42) | 5.1(3.69–6.61) | 8.25(5.75–10.78) | 12.58 | 1.2(0.92–1.48) |
| Burkina Faso | 6.53(4.78–8.73) | 25.67(19.07–33.96) | 13.11(9.59–17.23) | 22.76(17.05–29.16) | 100.8 | -0.44(-0.61–-0.28) |
| Burundi | 5.04(3.28–7.02) | 34.64(22.86–47.71) | 6.81(4.19–10.14) | 25.34(15.7–37.38) | 35.06 | -1.56(-1.74–-1.38) |
| Cabo Verde | 0.25(0.2–0.3) | 19(15.74–22.96) | 0.34(0.27–0.46) | 13.86(11.13–18.73) | 37.95 | -0.98(-1.31–-0.65) |
| Cambodia | 4.37(2.44–6.28) | 14.66(8.29–21.33) | 7.09(5.08–10.96) | 9.67(7.05–15.15) | 62.08 | -1.59(-1.69–-1.49) |
| Cameroon | 6.28(4.93–8.33) | 24.01(19–31.94) | 14.89(9.59–22.32) | 20.87(13.88–30.38) | 137.11 | -0.38(-0.54–-0.21) |
| Canada | 5.36(4.93–5.69) | 3.09(2.87–3.27) | 8.01(6.61–8.9) | 2.44(2.04–2.7) | 49.32 | -0.64(-0.77–-0.5) |
| Central African Republic | 2.52(1.63–3.44) | 33.74(22.56–45.49) | 4.21(2.52–6.28) | 29.31(18.31–43.24) | 66.89 | -0.57(-0.71–-0.43) |
| Chad | 3.77(2.9–5.24) | 23.88(18.33–33.13) | 7.64(5.28–10.28) | 25.08(17.7–33.2) | 102.82 | 0.34(0.21–0.46) |
| Chile | 10.08(8.41–10.69) | 17.97(14.88–19.11) | 9.11(8.14–10.36) | 7.19(6.42–8.29) | -9.57 | -3.46(-3.63–-3.28) |
| China | 264.2(205.24–435.27) | 5.85(4.59–9.57) | 534.41(303.99–688.56) | 5.13(2.92–6.6) | 102.28 | 0.09(-0.17–0.34) |
| Colombia | 16.12(15.02–17.9) | 16.09(14.99–18.07) | 23.95(18.39–31.2) | 8.41(6.41–10.99) | 48.57 | -2.73(-2.95–-2.5) |
| Comoros | 0.33(0.15–0.49) | 26.29(13.03–39.14) | 0.62(0.4–0.91) | 22.17(14.41–32.16) | 89.92 | -0.79(-0.97–-0.61) |
| Congo | 2.16(1.45–2.88) | 32.87(22.37–43.44) | 3.86(2.43–5.56) | 23.8(15.54–33.42) | 78.2 | -1.18(-1.35–-1.01) |
| Cook Islands | 0(0–0.01) | 5.88(4.36–8) | 0(0–0.01) | 3.91(2.77–5.01) | 28.42 | -1.01(-1.34–-0.69) |
| Costa Rica | 1.44(1.25–1.56) | 14.99(12.91–16.22) | 2.01(1.53–2.67) | 7.22(5.47–9.59) | 39.35 | -3.12(-3.49–-2.74) |
| Croatia | 2.54(2–2.81) | 7.1(5.67–7.86) | 1.64(1.23–2.12) | 3.68(2.77–4.75) | -35.34 | -2.11(-2.47–-1.74) |
| Cuba | 5.71(5.08–6.13) | 10.86(9.66–11.65) | 7.02(5.49–8.7) | 7.6(5.89–9.39) | 23.11 | -1.28(-1.36–-1.2) |
| Cyprus | 0.17(0.14–0.24) | 4.27(3.32–5.92) | 0.29(0.19–0.34) | 2.89(1.93–3.41) | 65.77 | -1.17(-1.37–-0.97) |
| Czechia | 6.39(5.95–6.76) | 8.61(8.06–9.12) | 4.44(3.57–5.43) | 4.35(3.52–5.41) | -30.51 | -2.4(-2.48–-2.31) |
| Côte d'Ivoire | 5.02(3.8–6.61) | 21.56(16.83–27.77) | 11.4(7.76–15.91) | 18.94(13.18–25.96) | 126.97 | -0.24(-0.35–-0.12) |
| Democratic People's Republic of Korea | 9.73(6.68–16.77) | 9.41(6.55–16.44) | 14.86(9.82–22.48) | 8.28(5.42–12.52) | 52.8 | -0.26(-0.37–-0.15) |
| Democratic Republic of the Congo | 25.06(16.98–33.46) | 25.35(17.41–34.49) | 48.72(31.45–68.59) | 21.84(14.12–31.24) | 94.41 | -0.45(-0.57–-0.34) |
| Denmark | 3.53(2.73–3.74) | 8.72(6.76–9.24) | 1.82(1.6–2.46) | 3.28(2.92–4.5) | -48.58 | -3.51(-3.83–-3.2) |
| Djibouti | 0.22(0.14–0.33) | 24.92(15.53–35.73) | 0.75(0.43–1.33) | 21.77(13–36.51) | 235.37 | -0.52(-0.64–-0.4) |
| Dominica | 0.08(0.07–0.1) | 22.15(18.32–26.88) | 0.08(0.06–0.1) | 17.33(13.33–22.01) | -8.98 | -0.88(-0.94–-0.81) |
| Dominican Republic | 2.56(2.16–3.29) | 12.29(10.4–15.26) | 6.58(4.76–9.15) | 13.31(9.61–18.49) | 156.95 | 0.51(0.38–0.63) |
| Ecuador | 5.16(4.39–5.96) | 17.73(15.08–20.4) | 10.57(7.95–13.98) | 13.3(10–17.5) | 104.71 | -0.79(-1.01–-0.57) |
| Egypt | 2.97(2.55–3.92) | 1.97(1.68–2.6) | 5.03(3.4–7.33) | 1.77(1.2–2.55) | 69.33 | -0.03(-0.14–0.09) |
| El Salvador | 2.9(2.61–3.77) | 17.17(15.43–22.43) | 5.23(3.77–6.94) | 15.26(10.99–20.32) | 80.03 | -1.07(-1.48–-0.65) |
| Equatorial Guinea | 0.34(0.2–0.5) | 27.31(16.07–39.74) | 0.54(0.33–0.9) | 17.09(10.9–26.96) | 57.7 | -1.76(-1.91–-1.61) |
| Eritrea | 2.26(1.39–3.49) | 30.94(18.72–47.65) | 5.51(3.48–8.05) | 30.26(19.35–43.43) | 143.85 | -0.01(-0.09–0.08) |
| Estonia | 1.18(1.02–1.27) | 9.8(8.47–10.57) | 0.68(0.52–0.88) | 5.08(3.77–6.59) | -42.16 | -2.61(-2.78–-2.43) |
| Eswatini | 0.43(0.28–0.6) | 23.24(15.27–32.38) | 0.97(0.5–1.66) | 26.85(13.98–45.7) | 127.61 | 1.07(0.41–1.72) |
| Ethiopia | 32.56(16.78–49.91) | 27.4(15.34–41.2) | 38.7(26.78–62.86) | 16.82(11.73–27.12) | 18.85 | -2.08(-2.26–-1.89) |
| Fiji | 0.58(0.29–0.76) | 26.79(13.39–34.7) | 0.9(0.37–1.23) | 22.1(9.26–29.74) | 54.7 | -0.25(-0.55–0.06) |
| Finland | 1.04(0.93–1.12) | 2.47(2.26–2.68) | 1.13(0.77–1.29) | 1.78(1.33–2.02) | 9.1 | -0.77(-0.98–-0.55) |
| France | 20.49(16.94–21.87) | 4.68(3.89–4.97) | 18.57(15.51–20.93) | 2.75(2.37–3.08) | -9.36 | -1.73(-1.89–-1.57) |
| Gabon | 0.76(0.53–1.02) | 23.98(16.75–32.29) | 1.02(0.66–1.48) | 16.73(11.04–24.33) | 35.07 | -1.35(-1.6–-1.1) |
| Gambia | 0.29(0.2–0.4) | 14.94(10.36–20.13) | 0.94(0.65–1.29) | 16.69(11.73–22.67) | 222.84 | 0.2(-0.01–0.41) |
| Georgia | 3.67(2.94–4.12) | 10.5(8.34–11.83) | 2.31(1.83–2.78) | 7.75(6.15–9.43) | -37.16 | -0.25(-0.88–0.38) |
| Germany | 33.41(30.58–35.78) | 4.98(4.61–5.26) | 26.09(23.03–28.95) | 2.87(2.57–3.16) | -21.9 | -2(-2.15–-1.85) |
| Ghana | 8.7(6.61–11.84) | 22.54(17.16–30.77) | 17.17(11.59–23.42) | 16.94(11.55–22.77) | 97.37 | -1.04(-1.12–-0.97) |
| Greece | 3.59(3.28–3.86) | 4.66(4.21–5.02) | 3.43(3.02–3.89) | 2.96(2.67–3.35) | -4.51 | -1.63(-1.82–-1.44) |
| Greenland | 0.03(0.03–0.04) | 17.22(13.88–20.95) | 0.03(0.02–0.04) | 9.1(6.99–11.85) | -13.23 | -2.77(-3–-2.53) |
| Grenada | 0.09(0.07–0.1) | 22.73(19.86–26.07) | 0.1(0.08–0.11) | 17(13.97–19.59) | 14.94 | -0.86(-1.15–-0.57) |
| Guam | 0.03(0.03–0.04) | 8.39(6.75–10.22) | 0.06(0.04–0.07) | 5.94(4.57–7.43) | 83.25 | -1.44(-1.9–-0.98) |
| Guatemala | 3.17(2.6–5.53) | 15.4(12.83–26.22) | 11.95(8.34–15.55) | 18.44(12.71–23.79) | 276.37 | 0.61(0.13–1.1) |
| Guinea | 7.62(5.84–9.58) | 42.09(32.63–53.22) | 11.49(8.24–15.28) | 36.16(26.43–47.56) | 50.71 | -0.39(-0.43–-0.35) |
| Guinea-Bissau | 0.82(0.54–1.17) | 32.43(21.5–46.02) | 1.41(0.88–1.98) | 29.28(18.87–40.39) | 71.13 | -0.14(-0.24–-0.04) |
| Guyana | 0.64(0.52–0.76) | 28.31(23.24–33.82) | 0.74(0.56–0.98) | 21.01(15.93–27.5) | 17.13 | -1.05(-1.2–-0.9) |
| Haiti | 7.8(3.3–10.44) | 39.7(17.45–51.88) | 12.33(5.66–18.32) | 27.76(12.82–40.33) | 58.07 | -1.12(-1.2–-1.04) |
| Honduras | 1.5(1.16–1.93) | 11.73(9.04–15.25) | 3.88(2.47–5.91) | 11.14(7.25–16.71) | 158.53 | -0.21(-0.38–-0.04) |
| Hungary | 6.81(6.39–7.32) | 8.79(8.24–9.49) | 4.81(3.87–5.98) | 5.18(4.13–6.51) | -29.3 | -1.99(-2.14–-1.83) |
| Iceland | 0.06(0.05–0.06) | 3.96(3.46–4.43) | 0.05(0.04–0.06) | 1.9(1.58–2.23) | -9.44 | -2.79(-2.99–-2.6) |
| India | 278.97(221.4–353.28) | 10.9(8.59–13.74) | 454.47(350.04–623.52) | 7.38(5.71–10.13) | 62.91 | -1.6(-1.81–-1.4) |
| Indonesia | 57.43(35.2–86.97) | 9.66(6.01–14.9) | 87.03(57.69–145.06) | 7.08(4.77–12.05) | 51.55 | -0.98(-1.09–-0.87) |
| Iran  (Islamic Republic of) | 4.16(2.97–5.01) | 3.22(2.33–4.05) | 7.61(5.52–8.74) | 2.06(1.49–2.37) | 82.94 | -1.64(-1.84–-1.44) |
| Iraq | 1.21(0.89–1.68) | 2.88(2.12–4.04) | 3.06(2.19–4.15) | 2.4(1.72–3.18) | 153.39 | -0.71(-0.84–-0.58) |
| Ireland | 0.86(0.79–0.97) | 4.25(3.9–4.69) | 1.01(0.82–1.17) | 2.81(2.2–3.26) | 17.76 | -1.18(-1.31–-1.06) |
| Israel | 0.76(0.69–0.93) | 3.01(2.75–3.65) | 1.5(1.18–1.7) | 2.53(1.99–2.85) | 97.76 | -0.79(-0.9–-0.68) |
| Italy | 9.06(8.52–11.09) | 1.94(1.83–2.36) | 15.89(9.7–17.71) | 2.23(1.38–2.47) | 75.34 | 1.04(0.8–1.29) |
| Jamaica | 1.62(1.39–1.75) | 17.66(15.07–19.07) | 2.47(1.87–3.15) | 15.9(11.93–20.5) | 52.3 | -0.44(-0.75–-0.12) |
| Japan | 33.94(31.11–37.35) | 3.66(3.37–4.06) | 42.1(33.48–46.88) | 2.78(2.18–3.04) | 24.06 | -0.67(-0.8–-0.53) |
| Jordan | 0.26(0.2–0.34) | 3.66(2.84–4.79) | 0.67(0.48–0.89) | 2.04(1.52–2.74) | 151.24 | -2.34(-2.58–-2.1) |
| Kazakhstan | 8.93(8.04–9.77) | 11.49(10.37–12.56) | 7.93(6.67–9.64) | 7.65(6.44–9.24) | -11.24 | -1.05(-1.39–-0.71) |
| Kenya | 5.95(4–9.62) | 12.55(8.53–19.92) | 17.25(11.61–27.99) | 12.92(8.82–20.82) | 189.76 | 0.38(0.2–0.55) |
| Kiribati | 0.19(0.15–0.24) | 83.8(64.61–106.92) | 0.29(0.21–0.37) | 69.52(51.09–88.92) | 53.01 | -0.64(-0.72–-0.56) |
| Kuwait | 0.09(0.07–0.11) | 3.19(2.51–3.76) | 0.21(0.16–0.29) | 1.76(1.3–2.41) | 129.26 | -1.4(-1.72–-1.08) |
| Kyrgyzstan | 2.25(1.88–2.44) | 12.66(10.65–13.76) | 2.52(2.03–2.98) | 8.94(7.19–10.54) | 12.26 | -1.14(-1.35–-0.93) |
| Lao People's Democratic Republic | 2.11(1.14–3.2) | 16.93(9.36–26.11) | 2.34(1.45–3.59) | 8.98(5.53–14.29) | 10.89 | -2.43(-2.52–-2.34) |
| Latvia | 1.76(1.48–1.91) | 8.32(7.1–9) | 0.95(0.71–1.28) | 4.46(3.29–6.09) | -46.4 | -2.21(-2.46–-1.96) |
| Lebanon | 0.47(0.35–0.59) | 3.94(3–4.95) | 0.7(0.51–0.97) | 2.43(1.77–3.39) | 47.83 | -1.76(-1.81–-1.7) |
| Lesotho | 1.14(0.76–1.66) | 20.16(13.48–29.14) | 2.78(1.4–4.75) | 35.96(18.42–60.81) | 144.49 | 3.25(2.72–3.77) |
| Liberia | 1.35(1.03–1.78) | 24.48(18.96–32.19) | 2.44(1.62–3.42) | 20.49(13.88–28.24) | 80.72 | -0.62(-0.72–-0.51) |
| Libya | 0.47(0.32–0.61) | 4.95(3.46–6.37) | 1.16(0.79–1.56) | 4.02(2.82–5.28) | 147.35 | -0.57(-0.81–-0.32) |
| Lithuania | 2.44(2.02–2.63) | 9.39(7.85–10.11) | 1.64(1.27–2.02) | 5.41(4.22–6.7) | -32.84 | -1.81(-2.01–-1.61) |
| Luxembourg | 0.13(0.11–0.14) | 4.3(3.78–4.72) | 0.1(0.08–0.12) | 1.94(1.63–2.42) | -20.43 | -2.66(-2.81–-2.5) |
| Madagascar | 8.01(5.52–10.57) | 26(17.73–33.81) | 15.74(10.1–22.9) | 21.87(14.01–31.45) | 96.54 | -0.69(-0.77–-0.62) |
| Malawi | 7.13(5.18–9.49) | 29.95(21.85–39.88) | 11.85(7.81–17.14) | 25.61(17.41–35.46) | 66.24 | -0.65(-0.85–-0.44) |
| Malaysia | 6.62(4.36–7.61) | 13.16(8.53–15.15) | 12.04(8.32–15.78) | 9(6.25–11.73) | 81.69 | -1.76(-2.02–-1.5) |
| Maldives | 0.06(0.02–0.09) | 13.12(5.71–18.24) | 0.06(0.05–0.08) | 4.14(3.25–5.28) | -0.36 | -4.54(-4.86–-4.22) |
| Mali | 5.81(4.37–7.2) | 24.3(18.6–30.21) | 8.94(6.21–12.29) | 18.42(13.08–24.83) | 53.95 | -1.15(-1.24–-1.07) |
| Malta | 0.08(0.07–0.09) | 3.39(2.96–3.78) | 0.08(0.06–0.1) | 1.78(1.48–2.15) | 1.24 | -1.87(-2.1–-1.64) |
| Marshall Islands | 0.02(0.01–0.03) | 22.73(15.66–35.38) | 0.04(0.03–0.07) | 21.42(12.97–33.44) | 102.85 | -0.26(-0.4–-0.12) |
| Mauritania | 1.56(1.13–2.07) | 27.94(20.38–37.02) | 2.02(1.42–2.79) | 18.07(12.89–24.55) | 29.2 | -1.26(-1.37–-1.15) |
| Mauritius | 0.41(0.37–0.45) | 9.97(8.88–10.95) | 0.54(0.42–0.68) | 5.76(4.54–7.29) | 30.95 | -2.41(-2.61–-2.21) |
| Mexico | 55.58(45.34–58.39) | 23.68(18.62–24.98) | 61.04(49.04–81.2) | 9.53(7.68–12.64) | 9.83 | -3.55(-3.74–-3.35) |
| Micronesia  (Federated States of) | 0.06(0.04–0.1) | 24.59(16.33–38.04) | 0.08(0.05–0.13) | 19.94(12.85–31.51) | 25.84 | -0.68(-0.83–-0.53) |
| Monaco | 0.01(0.01–0.02) | 4.02(2.85–5.17) | 0.01(0.01–0.02) | 2.69(1.96–3.6) | -13.83 | -1.27(-1.45–-1.09) |
| Mongolia | 1.1(0.86–1.42) | 18.7(14.62–23.65) | 1.75(1.26–2.43) | 12.2(8.77–16.57) | 59.63 | -1.99(-2.24–-1.74) |
| Montenegro | 0.17(0.15–0.23) | 5.12(4.26–6.79) | 0.23(0.19–0.3) | 4.84(3.89–6.13) | 32.49 | -0.27(-0.64–0.11) |
| Morocco | 6.85(4.55–8.42) | 9.12(6.13–11.13) | 13.34(8.74–17.93) | 7.83(5.31–10.43) | 94.62 | -0.45(-0.54–-0.35) |
| Mozambique | 10.42(6.83–14.73) | 28.06(18.5–39.66) | 20.43(12.68–30.29) | 28.76(18.43–41.69) | 96.1 | 0.29(0.05–0.54) |
| Myanmar | 20.9(12.12–33.22) | 15.07(8.95–24.35) | 21.45(14.99–36.12) | 7.73(5.48–13.24) | 2.61 | -2.57(-2.78–-2.37) |
| Namibia | 0.58(0.39–0.85) | 14.26(9.64–21.03) | 1.4(0.93–2.04) | 16.38(11.1–23.56) | 142.09 | 0.67(0.5–0.84) |
| Nauru | 0.01(0–0.01) | 23.31(16.41–34.61) | 0.01(0–0.01) | 19.09(11.85–28.07) | 5.82 | -0.58(-0.81–-0.35) |
| Nepal | 8.27(3.85–11.3) | 14.83(6.91–19.94) | 10.24(6.38–14.76) | 8.15(5.05–11.65) | 23.81 | -2.12(-2.54–-1.7) |
| Netherlands | 3.42(3.08–3.65) | 3.19(2.91–3.4) | 3.73(3.18–4.16) | 2.25(1.96–2.51) | 9.06 | -1.21(-1.33–-1.09) |
| New Zealand | 1.1(0.77–1.19) | 5.54(3.84–6) | 0.88(0.76–0.99) | 2.35(2.06–2.65) | -19.7 | -2.73(-3.14–-2.33) |
| Nicaragua | 2.07(1.62–2.33) | 21.76(17.02–24.61) | 4.2(3.36–5.69) | 16.74(13.48–22.26) | 103.17 | -1.24(-1.54–-0.93) |
| Niger | 4.12(3–5.67) | 25.33(18.56–34.5) | 10.77(7.44–14.84) | 23.62(16.62–31.87) | 161.63 | -0.36(-0.44–-0.28) |
| Nigeria | 31.14(21.21–45.37) | 14.07(9.66–20.53) | 64.36(42.83–93.05) | 12.08(8.26–16.87) | 106.69 | -0.39(-0.46–-0.33) |
| Niue | 0(0–0) | 15.01(11.21–22.41) | 0(0–0) | 10.9(7.36–16.14) | -33.1 | -1.25(-1.35–-1.15) |
| North Macedonia | 0.69(0.6–0.95) | 6.88(5.96–9.57) | 0.97(0.71–1.28) | 6.12(4.48–8.12) | 39.25 | -0.94(-1.38–-0.49) |
| Northern Mariana Islands | 0.02(0.02–0.03) | 21.67(16.4–27.95) | 0.04(0.03–0.05) | 15.64(11.99–20.08) | 87.69 | -0.97(-1.15–-0.78) |
| Norway | 1.64(1.5–1.73) | 4.98(4.65–5.24) | 1.31(1.15–1.47) | 2.8(2.52–3.12) | -19.68 | -2.02(-2.13–-1.91) |
| Oman | 0.15(0.1–0.2) | 4.59(3.16–6.34) | 0.23(0.17–0.29) | 3.1(2.37–3.8) | 58.22 | -0.94(-1.18–-0.71) |
| Pakistan | 13.49(10.87–16.92) | 4.64(3.69–5.77) | 29.47(20.95–41.39) | 4.55(3.23–6.28) | 118.38 | -0.35(-0.61–-0.09) |
| Palau | 0.02(0.01–0.03) | 39(28.36–53.71) | 0.03(0.02–0.04) | 29.79(21.68–39.14) | 54.98 | -0.84(-0.97–-0.72) |
| Palestine | 0.19(0.12–0.24) | 3.91(2.54–5.1) | 0.34(0.21–0.42) | 2.87(1.72–3.52) | 84.35 | -1.06(-1.31–-0.81) |
| Panama | 1.61(1.28–1.74) | 19.76(16–21.38) | 2.15(1.63–2.83) | 10.02(7.57–13.2) | 33.48 | -2.48(-2.66–-2.29) |
| Papua New Guinea | 1.48(0.88–2.45) | 14.19(8.84–23.91) | 3.97(2.29–6.02) | 14.2(8.49–22.27) | 168.28 | 0.17(0.09–0.25) |
| Paraguay | 2.36(1.85–2.79) | 18.82(14.87–22.27) | 5.06(3.62–6.75) | 16.69(12.02–22.19) | 113.89 | -0.66(-0.95–-0.38) |
| Peru | 11.99(10.02–14.78) | 18.01(15.11–22.03) | 20.83(14.74–28.05) | 12.23(8.68–16.51) | 73.78 | -1.59(-1.81–-1.37) |
| Philippines | 16.08(13.26–19.6) | 8.98(7.59–11.29) | 31.2(22.82–42.38) | 6.83(5.03–9.25) | 94.02 | -0.9(-1.1–-0.7) |
| Poland | 27.96(25.66–28.92) | 11.54(10.67–11.94) | 21.18(16.51–26.83) | 5.95(4.59–7.57) | -24.25 | -2.54(-2.68–-2.39) |
| Portugal | 4.47(4.08–4.83) | 6.24(5.67–6.7) | 3.74(3.28–4.24) | 3.17(2.82–3.58) | -16.41 | -2.5(-2.61–-2.38) |
| Puerto Rico | 1.02(0.92–1.11) | 5.27(4.77–5.77) | 1.24(0.95–1.6) | 3.77(2.82–4.96) | 21.44 | -1.15(-1.32–-0.98) |
| Qatar | 0.03(0.02–0.04) | 5.99(4.51–8.33) | 0.1(0.07–0.14) | 5.03(3.83–6.62) | 269.24 | -0.18(-0.47–0.11) |
| Republic of Korea | 11.4(9.98–14.8) | 6.04(5.45–8.27) | 12.7(10.1–15.87) | 2.72(2.17–3.4) | 11.35 | -3.47(-3.8–-3.14) |
| Republic of Moldova | 2.84(2.52–3.07) | 10.97(9.71–11.79) | 1.92(1.58–2.38) | 6.36(5.19–7.87) | -32.45 | -1.27(-1.55–-0.98) |
| Romania | 18.99(17.87–21.13) | 13.12(12.37–14.51) | 18.87(13.71–23.4) | 10.96(7.71–13.76) | -0.64 | -0.79(-1.03–-0.55) |
| Russian Federation | 70.87(64.65–77.23) | 6.36(5.86–6.98) | 68.45(53.05–84.2) | 5.6(4.22–6.95) | -3.41 | -0.6(-0.82–-0.37) |
| Rwanda | 6.93(4.78–9.36) | 37.11(25.71–49.62) | 8.08(5.36–12.05) | 20.62(14.1–29.74) | 16.63 | -2.82(-3.14–-2.5) |
| Saint Kitts and Nevis | 0.06(0.05–0.07) | 31.58(27.5–36.07) | 0.05(0.04–0.06) | 14.7(11.02–18.7) | -14.57 | -2.4(-2.57–-2.23) |
| Saint Lucia | 0.11(0.1–0.12) | 23.41(20.98–25.85) | 0.15(0.12–0.18) | 13.5(11.07–16.4) | 34.45 | -2.32(-2.61–-2.03) |
| Saint Vincent and the Grenadines | 0.11(0.1–0.12) | 27.65(24.59–30.82) | 0.13(0.11–0.16) | 20.61(17.18–24.38) | 24.85 | -1.22(-1.44–-0.99) |
| Samoa | 0.07(0.05–0.09) | 14.06(9.84–18.93) | 0.1(0.06–0.14) | 12.36(7.82–17.15) | 46.12 | -0.44(-0.54–-0.33) |
| San Marino | 0(0–0.01) | 2.27(1.79–2.99) | 0.01(0–0.01) | 2.35(1.44–3.62) | 90.98 | 0.74(0.46–1.02) |
| Sao Tome and Principe | 0.09(0.07–0.12) | 27.39(19.85–33.37) | 0.16(0.11–0.22) | 26.48(18.15–36.14) | 72.53 | -0.39(-0.62–-0.17) |
| Saudi Arabia | 0.75(0.52–1.24) | 2.85(2.04–4.59) | 2.04(1.49–2.79) | 2.35(1.73–3.11) | 170.24 | -0.56(-0.77–-0.36) |
| Senegal | 3.95(2.91–5.21) | 21.85(16.2–28.64) | 8.5(6.14–11.17) | 20.09(14.71–26.1) | 115.31 | -0.03(-0.23–0.17) |
| Serbia | 6.86(5.61–8.09) | 11.78(9.64–13.74) | 6.3(4.64–8.29) | 8.53(6.31–11.15) | -8.19 | -1.46(-1.69–-1.22) |
| Seychelles | 0.07(0.06–0.08) | 22.06(18.59–26.91) | 0.09(0.07–0.12) | 15.99(12.87–20.68) | 37.56 | -0.99(-1.06–-0.93) |
| Sierra Leone | 1.98(1.41–2.72) | 19.63(14.26–26.58) | 4.6(3.03–6.47) | 22.45(14.92–31.28) | 132.41 | 0.84(0.65–1.03) |
| Singapore | 0.98(0.86–1.06) | 7.57(6.73–8.18) | 1.05(0.92–1.23) | 2.62(2.27–3.06) | 8.09 | -4.04(-4.26–-3.81) |
| Slovakia | 2.25(1.94–2.63) | 6.99(6.01–8.18) | 2.4(1.59–3.13) | 5.19(3.39–6.74) | 6.78 | -0.79(-0.96–-0.63) |
| Slovenia | 0.83(0.62–1.09) | 6.04(4.46–7.92) | 0.62(0.45–0.83) | 2.89(2.13–3.92) | -25.65 | -2.78(-2.93–-2.62) |
| Solomon Islands | 0.25(0.11–0.43) | 32.01(16.3–56.36) | 0.59(0.27–0.91) | 29.44(15.37–45.45) | 135.74 | -0.18(-0.24–-0.11) |
| Somalia | 6.07(3.49–9.03) | 33.94(19.32–49.58) | 14.18(8.32–22.32) | 30.99(18.2–48.26) | 133.57 | -0.13(-0.2–-0.07) |
| South Africa | 22.88(17.41–29.74) | 17.44(13.2–23.07) | 44.15(36.44–52.17) | 16.64(13.75–19.58) | 93 | 0.12(-0.22–0.46) |
| South Sudan | 2.71(1.68–3.85) | 22.61(14.06–31.5) | 4.13(2.39–6.8) | 18.22(11.03–28.83) | 52.21 | -0.73(-0.82–-0.63) |
| Spain | 9.34(8.3–9.99) | 3.37(2.92–3.6) | 11.49(8.06–12.86) | 2.39(1.64–2.66) | 23.01 | -1.01(-1.18–-0.83) |
| Sri Lanka | 2.5(2.11–3.51) | 4.28(3.6–6.12) | 4.99(3.36–6.88) | 3.6(2.43–4.96) | 99.5 | -0.41(-0.71–-0.11) |
| Sudan | 2.21(1–3.02) | 4.51(2.04–6.07) | 3.18(1.94–4.65) | 3.27(2.13–4.62) | 43.49 | -1.08(-1.13–-1.03) |
| Suriname | 0.29(0.24–0.33) | 20.49(16.75–23.5) | 0.54(0.43–0.69) | 16.81(13.15–21.28) | 85.57 | -0.88(-1.1–-0.65) |
| Sweden | 3.02(2.76–3.4) | 4.01(3.7–4.58) | 2.78(2.43–3.08) | 2.72(2.43–3.02) | -7.8 | -1.35(-1.44–-1.25) |
| Switzerland | 2.44(1.7–2.65) | 4.35(3.08–4.72) | 1.96(1.62–2.2) | 2.17(1.87–2.45) | -20.02 | -2.37(-2.53–-2.22) |
| Syrian Arab Republic | 0.63(0.45–0.85) | 2.3(1.67–3.13) | 1.04(0.75–1.44) | 1.78(1.31–2.43) | 64.75 | -1.1(-1.27–-0.92) |
| Taiwan  (Province of China) | 10.12(9.13–10.66) | 12.93(11.77–13.64) | 10.33(7.99–13.93) | 5(3.86–6.83) | 2.05 | -4.13(-4.44–-3.82) |
| Tajikistan | 1.19(0.79–1.36) | 7.55(4.95–8.62) | 1.36(1.03–2.11) | 4.68(3.6–6.87) | 13.59 | -1.56(-1.97–-1.15) |
| Thailand | 31.36(23.32–36.82) | 14.76(11.1–17.29) | 36.57(26.68–52.33) | 6.73(4.9–9.6) | 16.62 | -3.27(-3.54–-3.01) |
| Timor-Leste | 0.22(0.13–0.33) | 12.47(7.66–18.71) | 0.38(0.25–0.59) | 9.03(6.06–14.06) | 70.18 | -1.3(-1.57–-1.03) |
| Togo | 1.91(1.5–2.52) | 23.87(18.91–31.46) | 4.75(3.4–6.5) | 20.1(14.66–26.99) | 149.05 | -0.49(-0.55–-0.43) |
| Tokelau | 0(0–0) | 23.19(16.24–35.44) | 0(0–0) | 15.89(11.13–22.7) | -34.19 | -1.32(-1.38–-1.26) |
| Tonga | 0.07(0.05–0.08) | 21.91(17.13–27.05) | 0.08(0.06–0.1) | 17.4(12.8–23.44) | 15.59 | -0.83(-0.97–-0.69) |
| Trinidad and Tobago | 0.83(0.76–0.9) | 18.47(16.92–20.18) | 1.09(0.81–1.44) | 11.55(8.59–15.34) | 31.36 | -2.03(-2.22–-1.84) |
| Tunisia | 0.92(0.67–1.13) | 3.55(2.61–4.37) | 1.69(1.16–2.31) | 2.57(1.77–3.5) | 84.15 | -1.14(-1.18–-1.1) |
| Turkey | 8.52(5.42–10.59) | 4.39(2.79–5.41) | 11.71(8–14.79) | 2.53(1.72–3.19) | 37.56 | -1.86(-2.28–-1.45) |
| Turkmenistan | 0.94(0.85–1.02) | 8.06(7.36–8.78) | 1.73(1.22–2.27) | 7.18(5.14–9.37) | 84.2 | 0.28(-0.11–0.66) |
| Tuvalu | 0.01(0.01–0.01) | 23.97(16.01–36.26) | 0.01(0.01–0.01) | 16.85(11.62–25.43) | -8.71 | -1.15(-1.24–-1.07) |
| Uganda | 8.66(6.04–11.57) | 23.15(16.47–30.47) | 22.22(15.77–29) | 24.29(17.61–30.9) | 156.7 | -0.1(-0.32–0.13) |
| Ukraine | 43.94(30.4–47.82) | 10.34(7.19–11.23) | 21.66(16.74–29.09) | 5.23(4.02–7.13) | -50.7 | -3.09(-3.32–-2.86) |
| United Arab Emirates | 0.17(0.12–0.24) | 12.26(7.64–17.88) | 0.76(0.54–1.07) | 6.8(4.3–9.12) | 349.67 | -1.75(-2.59–-0.89) |
| United Kingdom | 25.66(23.83–26.76) | 5.75(5.26–5.92) | 16.92(15.52–22.53) | 2.89(2.7–3.74) | -34.05 | -2.29(-2.56–-2.03) |
| United Republic of Tanzania | 16.9(11.45–22.73) | 26.72(18.09–35.33) | 33.33(22.74–46.49) | 22.51(15.48–30.9) | 97.19 | -0.52(-0.65–-0.39) |
| United States of America | 62.02(54.52–64.89) | 3.77(3.28–3.94) | 79.95(67.57–84.77) | 3.05(2.61–3.22) | 28.91 | -0.69(-0.83–-0.55) |
| United States Virgin Islands | 0.06(0.05–0.07) | 11.88(9.29–14.31) | 0.07(0.06–0.09) | 7.77(5.99–9.87) | 24.37 | -1.53(-1.58–-1.48) |
| Uruguay | 2.06(1.9–2.29) | 10.56(9.75–11.59) | 2.3(1.99–2.59) | 8.45(7.39–9.5) | 11.68 | -0.93(-1.1–-0.76) |
| Uzbekistan | 5.35(4.88–5.86) | 8.01(7.29–8.8) | 11.73(9.32–14.33) | 8.45(6.84–10.28) | 119.28 | 0.02(-0.23–0.26) |
| Vanuatu | 0.06(0.03–0.09) | 17.49(10.37–27.32) | 0.16(0.09–0.24) | 17.07(9.7–26.01) | 163.8 | -0.4(-0.6–-0.2) |
| Venezuela | 11.47(10.71–12.76) | 19.84(18.39–22.55) | 23.9(17.44–31.62) | 15.18(11.11–20.08) | 108.49 | -1.36(-1.58–-1.14) |
| Viet Nam | 24.78(18.46–33.23) | 10.59(7.92–14.24) | 47.18(32.73–61.61) | 8.8(6.11–11.45) | 90.42 | -0.63(-0.77–-0.48) |
| Yemen | 1.32(0.63–1.96) | 4.77(2.36–7.06) | 3.04(1.91–4.47) | 4(2.52–5.69) | 130.18 | -0.68(-0.75–-0.61) |
| Zambia | 6.1(4.24–8.01) | 34.99(24.98–45.63) | 11.36(7.56–15.99) | 26.42(17.91–37.04) | 86.37 | -1.37(-1.56–-1.18) |
| Zimbabwe | 6.54(4.61–8.34) | 28.55(20.45–36.44) | 14(9.37–19.49) | 31.39(21.68–43.63) | 114.13 | 1.46(0.95–1.97) |

EAPC: estimated annual percentage change; ASR, age–standardized rate; CI, confidence interval; UI: uncertainty interval.
