# Supplementary material for: Trends of cervical cancer at global, regional, and national level: data from the Global Burden of Disease study 2019
Source: BMC Public Health. 2021 May 12;21:894. doi: 10.1186/s12889-021-10907-5 (PMC8114503; doi:10.1186/s12889-021-10907-5)
Supplement: Supplementary file 10 — Additional file 10: Supplementary Table 6. the number and age-standardized rate of DALYs caused by cervical cancer at national level and both sexes in 1990 and 2019, and the percentage changes in number and the EAPCs from 1990 to 2019. [file 12889_2021_10907_MOESM10_ESM.doc]

**Supplementary Table 6**. the number and age-standardized rate of DALYs caused by cervical cancer at national level and both sexes in 1990 and 2019, and the percentage changes in number and the EAPCs from 1990 to 2019

|  | **1990** | | **2019** | | **1990-2019** | |
| --- | --- | --- | --- | --- | --- | --- |
| **Characteristics** | Number  ×10^3^ (95% UI) | ASR/100,000)  (95% UI) | Number  ×10^3^ (95% UI) | ASR/100,000)  (95% UI) | Changes in number (%) | EAPCs  (95%CI) |
| Afghanistan | 115.99(34–180.85) | 294.12(88.46–454.94) | 228.77(80.21–363.88) | 238.64(93.29–368.12) | 97.23 | -0.87(-1.07–-0.66) |
| Albania | 16.01(14.03–22.03) | 127.85(112.42–178.71) | 18.14(12.45–25.14) | 98.42(68.41–137.9) | 13.29 | -0.63(-0.82–-0.44) |
| Algeria | 179.42(117.58–233.97) | 239.74(161.58–310.59) | 265.55(182.18–369.81) | 133.16(92.95–182.64) | 48.01 | -2(-2.12–-1.88) |
| American Samoa | 0.4(0.31–0.54) | 281.62(225.86–383.14) | 0.76(0.57–0.98) | 287.21(217.27–372.02) | 89.83 | 0.15(-0.04–0.34) |
| Andorra | 0.45(0.31–0.62) | 155.75(109.9–217.62) | 0.78(0.54–1.07) | 122(85.23–168.61) | 74.22 | -0.91(-1.1–-0.73) |
| Angola | 241.94(156.96–354.54) | 862.06(563.78–1251.84) | 537.78(336.36–793.56) | 618.69(394.47–913.24) | 122.28 | -1.3(-1.46–-1.14) |
| Antigua and Barbuda | 1.05(0.91–1.22) | 381.14(329.41–442.08) | 1.74(1.41–2.14) | 308.72(250.89–377.22) | 65.43 | -0.93(-1.1–-0.75) |
| Argentina | 626.34(582.38–691.51) | 375.55(348.5–412.38) | 960.24(727.94–1068.19) | 366.91(275.7–408.93) | 53.31 | -0.19(-0.33–-0.05) |
| Armenia | 51.93(47.13–57.59) | 316.29(287.45–350.78) | 46.79(37.89–57.32) | 222.33(179.36–273.31) | -9.9 | -1.21(-1.46–-0.96) |
| Australia | 104.02(90.77–110.41) | 107.31(93.3–114.22) | 112.76(96.33–125.65) | 64.41(55.7–72.27) | 8.4 | -1.48(-1.9–-1.06) |
| Austria | 108.68(89.11–116.09) | 195.81(159.94–209.45) | 56.25(50.08–70.52) | 76.51(67.77–96.65) | -48.24 | -3.37(-3.61–-3.13) |
| Azerbaijan | 78.11(66.78–94.32) | 249.19(213.58–306.02) | 113.21(83.19–158.05) | 188.35(139.57–263.71) | 44.94 | -1.14(-1.31–-0.97) |
| Bahamas | 4.99(4.34–5.69) | 475.86(415.44–540.96) | 8.13(6.19–10.47) | 354.28(270.7–456.68) | 62.86 | -1.21(-1.34–-1.08) |
| Bahrain | 1.45(1.16–1.85) | 135.43(110.11–174.31) | 3.76(2.83–5.04) | 74.42(56.8–99.68) | 159.76 | -2.25(-2.44–-2.05) |
| Bangladesh | 1213.52(551.04–1593.89) | 433.38(194.25–567.09) | 1325.32(740.25–2046.63) | 180.42(100.78–277.14) | 9.21 | -2.81(-3.05–-2.58) |
| Barbados | 7.35(6.57–8.05) | 526.42(468.09–577.23) | 8.82(7.02–10.93) | 388.56(308.51–484.21) | 20 | -0.99(-1.07–-0.91) |
| Belarus | 185.41(171.28–203.25) | 263.05(243.22–289.11) | 147.2(109.25–206.4) | 194.83(142.45–272.77) | -20.61 | -1.42(-1.62–-1.21) |
| Belgium | 91.3(78.69–98.23) | 129.78(113.49–139.73) | 70.53(60.92–78.93) | 78.16(68.32–88.47) | -22.75 | -1.79(-1.93–-1.65) |
| Belize | 3.19(2.76–3.7) | 623.05(542.95–718.31) | 10.06(8.21–11.94) | 573.7(470.62–678.98) | 215.69 | -0.58(-0.95–-0.21) |
| Benin | 89.61(69.48–118.08) | 703.85(553.31–929.01) | 207.75(142.08–298.12) | 608.36(428.27–861.22) | 131.84 | -0.46(-0.51–-0.41) |
| Bermuda | 0.79(0.68–0.93) | 223.8(191.97–260.34) | 0.52(0.41–0.66) | 93.44(72.57–119.44) | -34.85 | -3.59(-3.84–-3.33) |
| Bhutan | 7.33(3.38–10.53) | 450.61(208.27–640.18) | 7.02(4.08–11.28) | 222.61(131.29–352.43) | -4.26 | -2.69(-2.94–-2.44) |
| Bolivia | 207.83(132.43–265.64) | 1022(659.2–1298.44) | 342.9(236.24–476.92) | 682.13(474.07–941.17) | 64.99 | -1.57(-1.7–-1.45) |
| Bosnia and Herzegovina | 48.67(42.74–58.13) | 197.44(174.25–236.66) | 46.66(31.85–61.08) | 179.4(120.09–235.69) | -4.14 | -0.43(-0.61–-0.25) |
| Botswana | 28.32(17.85–43.69) | 751.1(479.07–1147.11) | 80.03(45.68–125.85) | 810.03(468.54–1263.81) | 182.58 | 0.26(0–0.52) |
| Brazil | 2544.54(2401.26–2958.46) | 451.35(424.4–522.6) | 3484.16(3242.15–4042.98) | 268(249.59–309.88) | 36.93 | -2(-2.1–-1.89) |
| Brunei Darussalam | 3.99(2.89–5.04) | 546.1(411.3–674.07) | 6.34(4.98–8.3) | 305.13(245.47–395.91) | 58.77 | -1.98(-2.22–-1.74) |
| Bulgaria | 149.15(134.03–166.76) | 264.12(234.27–293.73) | 154.34(105.58–201.91) | 293.89(189.85–389.9) | 3.48 | 1.23(0.95–1.51) |
| Burkina Faso | 227.29(165.15–304.25) | 803.18(585.37–1080.76) | 465.61(331.64–619.93) | 698.95(507.68–922.64) | 104.86 | -0.54(-0.72–-0.37) |
| Burundi | 186.88(118.66–262.71) | 1176.27(756.52–1646.8) | 253.7(155.34–386.82) | 818.37(504.47–1231.79) | 35.76 | -1.77(-1.96–-1.58) |
| Cabo Verde | 7.14(5.93–8.98) | 559.7(465.85–722.66) | 9.12(7.16–13.49) | 365.13(288.33–535.6) | 27.71 | -1.31(-1.58–-1.04) |
| Cambodia | 160.73(87.72–228.09) | 486.73(270.54–694.94) | 237.32(167.21–357.27) | 305.98(215.02–460.08) | 47.65 | -1.77(-1.87–-1.67) |
| Cameroon | 224.34(175.66–299.05) | 759.22(592.81–999.16) | 532.64(329.45–821.28) | 635.16(403.6–955.34) | 137.43 | -0.56(-0.74–-0.37) |
| Canada | 159.14(148.86–169.44) | 96.74(90.36–103.67) | 210.79(178.1–234.25) | 77.31(66.46–86.75) | 32.45 | -0.58(-0.73–-0.43) |
| Central African Republic | 92.98(58.3–127.31) | 1114.49(713.49–1522.71) | 157.12(90.07–237.78) | 955.25(567.83–1427.5) | 68.98 | -0.61(-0.76–-0.47) |
| Chad | 128.76(97.89–175.89) | 751.69(571.04–1038.51) | 273.23(187.18–372.65) | 761.71(523.81–1026.84) | 112.21 | 0.18(0.04–0.32) |
| Chile | 327.87(283.19–348.23) | 552.07(473.26–586.5) | 252(223.82–300.05) | 212.58(188.53–253.96) | -23.14 | -3.58(-3.81–-3.35) |
| China | 8553.59(6544.84–14329.9) | 176.4(135.68–294.69) | 16222.42(8925.85–20908.64) | 157.5(86.9–202.91) | 89.66 | 0.16(-0.08–0.4) |
| Colombia | 555.43(517.9–616.14) | 495.77(463.64–547.54) | 736.02(553.02–966.06) | 265.5(199.02–347.9) | 32.51 | -2.58(-2.77–-2.39) |
| Comoros | 11.51(4.47–17.94) | 859.87(360.48–1318.84) | 20.96(12.92–31.34) | 707.02(443.12–1051.25) | 82.06 | -0.92(-1.16–-0.69) |
| Congo | 76.81(49.58–103.73) | 1068.34(711.52–1438.64) | 141.22(86.4–208.34) | 751.57(473.04–1086.01) | 83.86 | -1.28(-1.48–-1.08) |
| Cook Islands | 0.12(0.09–0.17) | 177.65(130.12–248.32) | 0.13(0.09–0.17) | 111.09(74.97–146.52) | 6.52 | -1.2(-1.55–-0.86) |
| Costa Rica | 47.61(41.67–51.39) | 453.33(396.84–489.77) | 61.5(45.51–82.52) | 222.04(164.39–298.15) | 29.18 | -2.97(-3.38–-2.56) |
| Croatia | 72.09(60.73–80.15) | 213.49(180.3–237.43) | 39.73(29.96–51.59) | 108.55(80.3–142.98) | -44.89 | -2.23(-2.57–-1.88) |
| Cuba | 189.63(164.44–204.73) | 356.95(309.72–385.12) | 203.43(155.67–254.54) | 242.78(184.66–304.63) | 7.28 | -1.52(-1.66–-1.39) |
| Cyprus | 4.62(3.72–6.69) | 110.53(88.92–158.84) | 6.9(4.67–8.21) | 72.28(49.31–85.95) | 49.28 | -1.38(-1.51–-1.25) |
| Czechia | 187(175.43–198.1) | 274.53(256.81–291.3) | 114.01(90.77–142.09) | 132.81(105.37–168.05) | -39.03 | -2.55(-2.64–-2.45) |
| Côte d'Ivoire | 190.61(140.26–253.58) | 672.74(511.79–879.95) | 414.31(275.04–591.89) | 569.24(385.3–795.51) | 117.36 | -0.38(-0.5–-0.25) |
| Democratic People's Republic of Korea | 335.65(220.99–582.51) | 312.19(207.22–539.82) | 482.47(305.51–722.14) | 279.56(175.29–415.39) | 43.74 | -0.2(-0.31–-0.1) |
| Democratic Republic of the Congo | 908.56(602.45–1223.79) | 811.92(550.41–1085.86) | 1726.85(1105.2–2422.81) | 683.21(440.04–957.89) | 90.06 | -0.54(-0.66–-0.41) |
| Denmark | 93.86(73.1–99.69) | 265.23(206.22–282.92) | 42.48(37.68–58.68) | 94.17(82.88–128.45) | -54.73 | -3.75(-4.09–-3.42) |
| Djibouti | 8.56(5.04–13) | 800.6(488.78–1178.7) | 27.84(15.29–50.71) | 675.46(387.57–1185.74) | 225.35 | -0.66(-0.79–-0.53) |
| Dominica | 2.35(1.92–2.83) | 706.62(573.73–861.25) | 2.13(1.61–2.76) | 535.72(401.54–695.38) | -9.02 | -1.14(-1.2–-1.07) |
| Dominican Republic | 91.25(76.3–118.94) | 381.81(320.92–497.22) | 209.13(144.33–294.35) | 405.51(281.44–573.79) | 129.19 | 0.33(0.18–0.47) |
| Ecuador | 173.28(148.21–202.25) | 535.57(453.8–621.31) | 318.94(239.23–435.45) | 383.79(286.38–523.03) | 84.06 | -0.97(-1.22–-0.73) |
| Egypt | 95.29(80.38–121.86) | 54.39(46.62–70.55) | 154.8(103.85–227.52) | 45.13(30.67–65.66) | 62.45 | -0.35(-0.45–-0.25) |
| El Salvador | 100.22(89.36–129.26) | 559.15(499.1–721.63) | 155.92(110.46–212.27) | 463.1(328.34–631.33) | 55.58 | -1.3(-1.7–-0.91) |
| Equatorial Guinea | 12.42(7.27–18.07) | 899.27(526.59–1309.48) | 19.15(10.97–32.82) | 508.3(302.02–846.19) | 54.25 | -2.12(-2.28–-1.95) |
| Eritrea | 86.34(52.7–133.63) | 1047.27(639.22–1606.41) | 201.27(127.48–296.94) | 973.58(614.4–1424.77) | 133.12 | -0.15(-0.22–-0.08) |
| Estonia | 33.88(29.44–36.66) | 307.74(265.34–334.5) | 17.35(12.94–22.57) | 160.82(119.98–213.43) | -48.8 | -2.68(-2.87–-2.49) |
| Eswatini | 14.8(9.58–21.05) | 710.49(459.87–1006.76) | 32.13(16.17–55.77) | 794.74(400.57–1360.64) | 117.16 | 1.01(0.32–1.7) |
| Ethiopia | 1244.88(582.85–1926.06) | 914.79(470.11–1411.45) | 1335.8(908.61–2196.07) | 497.94(343.9–810.21) | 7.3 | -2.56(-2.77–-2.36) |
| Fiji | 21.53(10.69–28.59) | 839.53(413.1–1108.9) | 30.09(12.44–41.7) | 680.13(283.37–939.18) | 39.76 | -0.28(-0.59–0.03) |
| Finland | 24.8(22.87–27.52) | 67.2(61.76–75.82) | 23.64(17.76–26.9) | 47.49(38.57–54.33) | -4.68 | -0.92(-1.07–-0.76) |
| France | 527.51(447.4–562.48) | 139.2(118.87–148.44) | 426.86(367.19–481.29) | 82.27(71.37–93.13) | -19.08 | -1.71(-1.85–-1.57) |
| Gabon | 24.6(16.97–33.37) | 755.24(518.24–1021.93) | 34.18(21.51–50.48) | 500.16(318.3–731.05) | 38.93 | -1.53(-1.82–-1.24) |
| Gambia | 10.52(6.99–14.74) | 465.77(310.54–647.99) | 33.05(22.07–47.01) | 523.79(356.56–732.28) | 214.29 | 0.19(-0.04–0.42) |
| Georgia | 119.67(91.59–136.11) | 358.06(272.58–408.23) | 69.25(54.4–84.61) | 261.64(203.26–320.31) | -42.13 | -0.39(-0.97–0.2) |
| Germany | 909.38(841.66–958.82) | 157.76(139.76–167.13) | 610.56(545.69–677.13) | 86.56(77.18–97.32) | -32.86 | -2.16(-2.28–-2.05) |
| Ghana | 323.8(238.49–440.96) | 733.5(550.81–1000.45) | 602.48(398.56–842.5) | 516.85(344.09–709.92) | 86.06 | -1.29(-1.38–-1.2) |
| Greece | 94.85(85.7–102.05) | 134.6(120.52–144.84) | 77.24(69.66–86.95) | 86.06(77.46–96.73) | -18.57 | -1.53(-1.78–-1.27) |
| Greenland | 1.24(0.99–1.55) | 567.44(455.23–700.88) | 0.97(0.73–1.28) | 298.21(224.13–393.34) | -21.98 | -2.65(-2.82–-2.48) |
| Grenada | 2.56(2.22–2.95) | 746.63(643.95–864.73) | 3.02(2.48–3.53) | 525.91(427.55–613.68) | 17.65 | -1.09(-1.38–-0.81) |
| Guam | 1.02(0.83–1.27) | 222.88(182.37–275.55) | 1.72(1.31–2.18) | 187.8(142.79–236.93) | 68.98 | -0.82(-1.19–-0.44) |
| Guatemala | 116.12(93.5–209.27) | 484.16(394.89–855.1) | 393.91(281.15–519.11) | 559.06(395–734.61) | 239.23 | 0.46(0.01–0.92) |
| Guinea | 264.95(202.59–332.76) | 1351.39(1033.4–1701.11) | 412.8(292.73–560.37) | 1143.8(813.77–1527.84) | 55.8 | -0.43(-0.49–-0.38) |
| Guinea-Bissau | 31.24(20.31–45.09) | 1085.85(707.95–1560.41) | 52.94(32.7–75.81) | 937.63(586.74–1325.59) | 69.46 | -0.31(-0.42–-0.2) |
| Guyana | 22.98(18.52–27.98) | 910.53(741.94–1104.08) | 25.73(18.84–34.73) | 683.14(502.45–914.26) | 11.97 | -1.04(-1.23–-0.86) |
| Haiti | 296.39(114.11–409.7) | 1358.26(558.52–1843.94) | 463.81(211.62–696.76) | 913.06(419.09–1364.03) | 56.49 | -1.23(-1.33–-1.14) |
| Honduras | 59.77(45.45–76.44) | 420.25(322.77–541.17) | 131.9(79.66–207.45) | 343.73(210.79–530.99) | 120.66 | -0.8(-0.94–-0.66) |
| Hungary | 206.1(192.8–224.37) | 291.99(271.32–317.76) | 133.42(105.92–168.41) | 170.06(132.84–217.74) | -35.27 | -2.12(-2.27–-1.96) |
| Iceland | 1.61(1.4–1.8) | 120.09(103.81–134.65) | 1.28(1.06–1.5) | 54.93(45.72–64.42) | -20.58 | -3.01(-3.2–-2.81) |
| India | 10292.21(8087.15–12909.48) | 355.76(281.53–447.7) | 15544.89(11982.23–20941.37) | 239.49(184.74–325.15) | 51.04 | -1.6(-1.81–-1.38) |
| Indonesia | 2095.75(1256.59–3108.11) | 316.01(191.9–475.38) | 2919.25(1901.14–4659.41) | 216.87(143.05–352.41) | 39.29 | -1.22(-1.36–-1.08) |
| Iran  (Islamic Republic of) | 139.87(98.3–162.21) | 90.42(63.85–107.54) | 223.67(161.99–257.05) | 54.11(38.94–62.2) | 59.92 | -1.9(-2.09–-1.71) |
| Iraq | 39.44(28.57–55.01) | 85.55(61.93–118) | 103.07(71.48–142.11) | 70.2(49.79–95.2) | 161.3 | -0.77(-0.91–-0.64) |
| Ireland | 26.17(23.3–28.74) | 138.91(121.54–152.81) | 29.8(21.67–34.95) | 90.3(63.79–105.93) | 13.89 | -1.2(-1.35–-1.06) |
| Israel | 21.67(19.77–25.65) | 87.98(80.08–104.66) | 38.72(30.08–43.79) | 72.51(56.03–82.16) | 78.68 | -0.83(-0.94–-0.72) |
| Italy | 237.17(225.57–284.56) | 57.43(54.58–69.29) | 357.97(220.68–396.78) | 64.96(40.66–71.73) | 50.93 | 1(0.75–1.25) |
| Jamaica | 48.26(41.89–52.62) | 545.65(469.67–593.95) | 77.77(57.84–101.83) | 511.65(379.85–672.19) | 61.14 | -0.26(-0.59–0.06) |
| Japan | 922.62(872.62–1036.95) | 104.7(99.43–117.74) | 974.54(763.68–1068.71) | 90.99(67.46–99.86) | 5.63 | -0.13(-0.25–-0.02) |
| Jordan | 9.09(6.89–11.66) | 106.21(81.44–135.46) | 21.48(15.14–28.75) | 55.25(39.79–73.64) | 136.23 | -2.6(-2.84–-2.35) |
| Kazakhstan | 279.78(255.83–309.94) | 355.99(325.64–393.77) | 276.46(229.6–336) | 262.01(217.61–317.76) | -1.18 | -0.66(-1.01–-0.3) |
| Kenya | 206.91(136.84–341.69) | 390.27(258.53–636.44) | 604.21(406.7–987.44) | 395.3(266.45–641.69) | 192.01 | 0.29(0.1–0.47) |
| Kiribati | 6.95(5.14–8.99) | 2728.8(2080.98–3506.63) | 10.38(7.36–13.6) | 2143.06(1534.43–2781.26) | 49.46 | -0.84(-0.93–-0.76) |
| Kuwait | 3.34(2.73–3.95) | 92.15(73.96–109.07) | 7.09(5.31–10.35) | 44.34(33.14–62.29) | 112.14 | -2.11(-2.41–-1.8) |
| Kyrgyzstan | 72.72(63.06–79.47) | 411.64(360.62–449.93) | 87.58(69.92–104.6) | 291.78(233.19–347.31) | 20.44 | -1.14(-1.36–-0.91) |
| Lao People's Democratic Republic | 76.99(38.72–115.22) | 572.86(300.19–865.17) | 83.81(51.52–124.54) | 289.76(180.22–437.83) | 8.86 | -2.59(-2.68–-2.5) |
| Latvia | 49.54(42.81–53.78) | 255.6(225.76–278.21) | 23.03(16.96–31.59) | 134.7(96.35–188.74) | -53.52 | -2.38(-2.66–-2.11) |
| Lebanon | 15.6(11.01–20.14) | 120.71(87.07–154.25) | 20.69(13.98–28.72) | 71.83(48.58–99.59) | 32.65 | -1.92(-2.03–-1.81) |
| Lesotho | 35.22(22.79–52.8) | 600.41(390.88–894.47) | 90.28(44.34–156.55) | 1087.77(542.05–1883.99) | 156.32 | 3.44(2.87–4.02) |
| Liberia | 45.65(34.25–61.22) | 765.1(578.56–1022.04) | 88.19(57.68–125.32) | 620.59(413.31–875.05) | 93.18 | -0.79(-0.91–-0.68) |
| Libya | 16.07(10.81–20.96) | 155.43(105.53–201.39) | 40.55(25.26–55.27) | 124.02(82.41–167.15) | 152.34 | -0.67(-0.93–-0.4) |
| Lithuania | 71.27(60.19–77) | 293.7(249.87–317.45) | 40.91(31.85–50.97) | 166.86(126.83–212.66) | -42.6 | -1.92(-2.15–-1.68) |
| Luxembourg | 3.4(2.97–3.73) | 129.49(112.17–142.3) | 2.49(2.09–3.11) | 55.34(46.35–69.27) | -26.83 | -2.89(-3.04–-2.74) |
| Madagascar | 312.05(217.91–417.37) | 910.14(627.39–1208.71) | 597.82(385.71–877.12) | 718.9(462.52–1047.43) | 91.58 | -0.88(-0.94–-0.83) |
| Malawi | 264.61(188.15–355.36) | 988.47(712.56–1319.25) | 428.65(269.44–645.01) | 820.33(531.31–1198.96) | 61.99 | -0.77(-1.01–-0.54) |
| Malaysia | 213.52(144.7–246.86) | 376.97(251.89–432.9) | 353.86(251.89–471.08) | 243.91(174.02–323.1) | 65.73 | -1.89(-2.18–-1.59) |
| Maldives | 2.2(0.88–3.15) | 410.91(169.95–589.38) | 1.85(1.42–2.46) | 110.48(85.88–144.15) | -15.6 | -5.06(-5.40–-4.72) |
| Mali | 210.9(157.71–264.22) | 804.34(604.91–1000.67) | 319.45(219.44–448.88) | 580.52(400.89–803.01) | 51.47 | -1.36(-1.47–-1.26) |
| Malta | 2.25(1.97–2.52) | 99.08(86.33–110.42) | 1.86(1.55–2.26) | 50.94(42.29–61.79) | -17.34 | -1.9(-2.15–-1.66) |
| Marshall Islands | 0.78(0.52–1.17) | 725.03(496.46–1113.53) | 1.65(0.92–2.6) | 696.44(400.6–1093.95) | 110.87 | -0.2(-0.36–-0.03) |
| Mauritania | 52.26(36.82–69.48) | 872.15(617–1155.34) | 65.65(44.31–93.53) | 523.83(357.58–733.13) | 25.64 | -1.53(-1.62–-1.43) |
| Mauritius | 12.58(11.22–13.92) | 286.06(255.78–316.62) | 14.82(11.58–19.16) | 162.86(126.99–211.25) | 17.81 | -2.49(-2.7–-2.28) |
| Mexico | 1813.15(1561.82–1900.77) | 676.78(568.44–710.47) | 1889.21(1507.15–2549.78) | 282.99(226.19–380.52) | 4.19 | -3.39(-3.6–-3.18) |
| Micronesia  (Federated States of) | 2.23(1.4–3.49) | 787.61(508.17–1228.66) | 2.75(1.56–4.44) | 622.06(363.15–1000.78) | 22.83 | -0.76(-0.89–-0.63) |
| Monaco | 0.33(0.23–0.42) | 122.28(86.99–157.36) | 0.27(0.19–0.36) | 80.39(57.89–112.07) | -17.5 | -1.34(-1.54–-1.14) |
| Mongolia | 36.24(28.03–48.98) | 583.47(452.93–774.4) | 60.72(42.95–87.22) | 363.49(262.7–509.99) | 67.54 | -2.19(-2.45–-1.93) |
| Montenegro | 5.88(4.84–7.62) | 174.02(142.76–225.2) | 7.03(5.57–8.79) | 161.37(127.8–201.84) | 19.47 | -0.39(-0.81–0.04) |
| Morocco | 229.67(149.25–284.87) | 281.05(185.51–345.58) | 431.03(262.51–601.39) | 236.51(150.1–326.2) | 87.68 | -0.55(-0.66–-0.45) |
| Mozambique | 381.83(242.89–551.1) | 918.05(596.76–1303.75) | 740.55(444.22–1131.27) | 915.04(561.97–1372.26) | 93.95 | 0.22(-0.06–0.49) |
| Myanmar | 777.56(435.79–1209.36) | 521.01(297.92–814.26) | 724.15(489.23–1169.11) | 247.28(167.17–404.93) | -6.87 | -2.88(-3.12–-2.64) |
| Namibia | 18.71(12.35–27.62) | 437.88(292.34–650.12) | 44.86(28.58–67.36) | 486.53(316.49–722.51) | 139.74 | 0.58(0.41–0.75) |
| Nauru | 0.21(0.13–0.32) | 737.61(489.42–1115.4) | 0.23(0.13–0.36) | 612.18(356.11–930.95) | 7.65 | -0.53(-0.78–-0.27) |
| Nepal | 312.54(144.5–433.39) | 508.47(236.17–695.19) | 351.81(218.05–509.94) | 259.06(161.28–373.19) | 12.56 | -2.38(-2.82–-1.94) |
| Netherlands | 92.25(84.23–98.38) | 96.07(87.96–102.81) | 87.57(77.15–97.99) | 65.34(57.67–73.69) | -5.07 | -1.4(-1.52–-1.27) |
| New Zealand | 35.33(23.05–38.45) | 188.04(121.21–205.4) | 23.02(20.45–25.98) | 71.08(62.75–80.39) | -34.85 | -3.16(-3.6–-2.71) |
| Nicaragua | 76.41(60.65–86.62) | 719.61(569.81–808.62) | 129.08(99.49–175.18) | 465.42(359.89–626.32) | 68.92 | -1.68(-1.83–-1.52) |
| Niger | 153.23(110.55–212.43) | 803.87(585.5–1111.26) | 376.36(251.48–530.52) | 711.53(487.57–984.2) | 145.61 | -0.62(-0.72–-0.52) |
| Nigeria | 1034.34(688.37–1550.91) | 436.68(292.44–646.45) | 2254.46(1477.07–3320.52) | 359.84(239.42–520.17) | 117.96 | -0.57(-0.63–-0.51) |
| Niue | 0.05(0.04–0.07) | 458.28(329.31–685.56) | 0.03(0.02–0.05) | 333.96(213.73–501.51) | -30.34 | -1.27(-1.37–-1.16) |
| North Macedonia | 23.74(20.52–31.8) | 227.25(196.69–305.26) | 28.86(20.76–38.54) | 190.84(137.26–255.65) | 21.59 | -1.2(-1.66–-0.75) |
| Northern Mariana Islands | 0.88(0.62–1.19) | 657.31(486.25–871.08) | 1.3(0.96–1.7) | 461.63(341.68–603.48) | 48.03 | -1.07(-1.27–-0.88) |
| Norway | 43.11(40.39–45.39) | 155.93(145.18–164.12) | 31.3(28.37–34.9) | 80.43(73.11–90.05) | -27.39 | -2.34(-2.43–-2.25) |
| Oman | 4.86(3.3–6.69) | 131.57(89.5–180.26) | 7.64(5.61–9.65) | 78.01(58.73–96.62) | 57.18 | -1.45(-1.76–-1.14) |
| Pakistan | 483.96(390.53–607.9) | 151.8(122.5–191.67) | 1107.86(782.03–1580.9) | 149.93(107.17–210.77) | 128.92 | -0.35(-0.63–-0.06) |
| Palau | 0.69(0.49–0.96) | 1188.69(843.68–1638.67) | 1.02(0.73–1.37) | 896.44(641.92–1196) | 47.37 | -0.88(-1.02–-0.75) |
| Palestine | 5.34(3.45–7.31) | 101.84(65.98–137.04) | 9.78(6.52–12.1) | 69.83(44.73–86.02) | 83.11 | -1.28(-1.56–-1) |
| Panama | 57.43(43.35–62.77) | 657.79(502.43–716.55) | 67.91(49.57–89.96) | 321.04(234.3–425.46) | 18.26 | -2.63(-2.9–-2.36) |
| Papua New Guinea | 55.31(31.92–88.22) | 454.62(269.17–752.37) | 152(85.21–227.35) | 451.63(262.37–687.26) | 174.81 | 0.17(0.08–0.27) |
| Paraguay | 83.42(64.01–97.86) | 620.61(471.98–728.89) | 168.59(118.25–228.05) | 535.09(376.32–724.12) | 102.1 | -0.79(-1.04–-0.53) |
| Peru | 411.39(339.5–509.28) | 566.72(470.34–700.28) | 634.1(434.11–878.06) | 366.03(250.6–507.31) | 54.14 | -1.78(-2–-1.55) |
| Philippines | 597.76(467.91–710.63) | 292.84(238.34–356.17) | 1105.66(788.47–1477.99) | 226.75(164.63–303.9) | 84.97 | -0.82(-1–-0.64) |
| Poland | 847.55(778.56–877.86) | 366.94(339.3–380.21) | 553(430.01–703.66) | 178.24(138.08–229.12) | -34.75 | -2.76(-2.93–-2.59) |
| Portugal | 127.87(115.13–137.47) | 195.42(173.16–210.85) | 90.5(79.33–103.24) | 96.32(83.79–110.44) | -29.23 | -2.69(-2.85–-2.52) |
| Puerto Rico | 31.18(28.46–34.32) | 162.58(148.18–178.84) | 33.1(24.72–43.58) | 124.59(91.04–165.92) | 6.17 | -0.93(-1.11–-0.75) |
| Qatar | 0.99(0.72–1.4) | 158.1(118.14–218.37) | 3.62(2.58–5.14) | 104.18(78.22–139.27) | 264.9 | -1.21(-1.46–-0.97) |
| Republic of Korea | 411.24(342.72–480.92) | 201.47(172.86–243.22) | 326.73(267.9–412.87) | 77.9(63.89–97.95) | -20.55 | -3.91(-4.17–-3.64) |
| Republic of Moldova | 93.24(79.2–100.72) | 364.28(308.33–392.38) | 60.49(48.76–74.36) | 218.96(174.7–270.94) | -35.12 | -1.16(-1.44–-0.87) |
| Romania | 637.75(597.74–680.58) | 462.62(432.93–492.65) | 545.32(376.21–692.95) | 364.48(249.97–466.88) | -14.49 | -1.09(-1.36–-0.82) |
| Russian Federation | 1998.73(1877.05–2205.54) | 192.57(180.52–213.93) | 2173.05(1622.63–2703.7) | 199.87(145.46–250.63) | 8.72 | 0.03(-0.25–0.32) |
| Rwanda | 260.4(174.21–359.54) | 1274.62(872.59–1746.89) | 283.44(183.54–435.02) | 643.91(423.28–974.37) | 8.85 | -3.25(-3.61–-2.88) |
| Saint Kitts and Nevis | 1.81(1.54–2.07) | 1028.25(871.33–1179.37) | 1.53(1.02–2.06) | 415.73(276.56–564.04) | -15.67 | -3.09(-3.29–-2.88) |
| Saint Lucia | 3.69(3.27–4.1) | 750.89(666.1–835.56) | 4.73(3.8–5.84) | 426.97(343.36–525.68) | 28.15 | -2.24(-2.5–-1.98) |
| Saint Vincent and the Grenadines | 3.39(2.99–3.82) | 879.84(771.26–996.01) | 4.2(3.46–5.05) | 653.61(538.29–781.93) | 24.05 | -1.36(-1.56–-1.16) |
| Samoa | 2.29(1.5–3.11) | 460.95(307.02–624.18) | 3.35(1.97–4.88) | 409.14(244.4–590.47) | 46.57 | -0.38(-0.48–-0.28) |
| San Marino | 0.1(0.07–0.13) | 63.2(48.61–83.73) | 0.18(0.11–0.28) | 68.4(40.94–108.48) | 83.05 | 0.85(0.58–1.12) |
| Sao Tome and Principe | 3.04(2.07–3.83) | 847.69(593.07–1063.39) | 5.66(3.95–7.88) | 806.2(560.01–1102.19) | 86.2 | -0.53(-0.82–-0.24) |
| Saudi Arabia | 24.86(16.81–43.37) | 77.97(53.93–131.98) | 77.25(54.94–109.56) | 64.92(48.08–88.08) | 210.72 | -0.46(-0.66–-0.25) |
| Senegal | 139.48(99.52–186.29) | 685.36(495.91–912.18) | 282.14(196.76–379.89) | 594.14(423.11–792.84) | 102.28 | -0.29(-0.49–-0.09) |
| Serbia | 221.54(179.36–257.22) | 383.66(310.16–441.17) | 176.56(128.2–230.98) | 272.05(196.42–359.46) | -20.3 | -1.58(-1.83–-1.33) |
| Seychelles | 2.03(1.7–2.43) | 691.78(576.6–828.32) | 2.76(2.18–3.62) | 468.49(371.07–612.83) | 36.44 | -1.17(-1.26–-1.09) |
| Sierra Leone | 67.08(46.45–92.5) | 604.71(422.63–839.9) | 163.27(105–232.01) | 691.28(450.32–974.43) | 143.39 | 0.83(0.62–1.03) |
| Singapore | 31.98(28.26–34.95) | 225.26(198.11–244.91) | 28.79(24.89–34.23) | 71.95(62.24–85.34) | -9.98 | -4.31(-4.55–-4.07) |
| Slovakia | 71.74(61.94–82.77) | 233.19(201.06–269.02) | 70.48(45.64–92.59) | 170.3(110.45–225.67) | -1.76 | -0.86(-1.02–-0.7) |
| Slovenia | 24.13(17.74–32.5) | 188.17(137.56–256.08) | 15(11.01–20.54) | 87.45(62.74–121.35) | -37.84 | -2.97(-3.16–-2.78) |
| Solomon Islands | 9.98(4.13–16.84) | 1114.26(502.39–1911.87) | 23.83(9.51–37.34) | 1018.69(457.59–1570.95) | 138.8 | -0.16(-0.23–-0.09) |
| Somalia | 236.2(135.84–354.6) | 1151.3(663.13–1709.34) | 527.69(307.41–825.23) | 1013.76(591.13–1575.14) | 123.41 | -0.28(-0.34–-0.23) |
| South Africa | 847.11(648.77–1067.51) | 597.49(458.25–764.65) | 1410.35(1148.27–1679.31) | 500.44(407.83–596.02) | 66.49 | -0.28(-0.6–0.05) |
| South Sudan | 97.58(59.15–143.36) | 725.75(446.17–1041.08) | 150.97(83.57–258.51) | 566.64(328.63–932) | 54.71 | -0.84(-0.96–-0.73) |
| Spain | 260.16(220.88–278.04) | 104.73(87.61–111.95) | 278.38(189.01–310.81) | 71.2(49.51–79.92) | 7 | -1.24(-1.38–-1.09) |
| Sri Lanka | 85.61(71.43–115.74) | 128.96(108.39–178) | 140.7(94.38–196.08) | 101(67.8–140.67) | 64.35 | -0.71(-0.98–-0.44) |
| Sudan | 76.21(34.33–106.37) | 137.69(61.93–189.52) | 109.65(63.2–168.07) | 93.66(56.31–137.99) | 43.88 | -1.3(-1.35–-1.25) |
| Suriname | 9.85(7.84–11.37) | 651.05(527.37–750.78) | 17.61(13.63–22.34) | 543.46(419.2–691.2) | 78.89 | -0.91(-1.14–-0.68) |
| Sweden | 73.14(67.94–84.2) | 118.69(109.99–136.88) | 59.94(53.89–66.51) | 75.64(68.77–84.65) | -18.05 | -1.48(-1.56–-1.39) |
| Switzerland | 60.64(44.4–65.94) | 124.18(93.66–135.3) | 42.62(36.89–48.4) | 58.09(50.73–66.63) | -29.7 | -2.68(-2.83–-2.52) |
| Syrian Arab Republic | 22.18(15.4–29.62) | 68.49(48.28–90.74) | 31.9(22.37–44.99) | 46.56(33.24–64.92) | 43.79 | -1.54(-1.77–-1.3) |
| Taiwan  (Province of China) | 321.83(281.69–339.84) | 380.08(336.13–401.34) | 258.33(194.84–361.79) | 133.34(100.83–187.46) | -19.73 | -4.43(-4.76–-4.11) |
| Tajikistan | 39.86(28.32–45.78) | 243.88(170.22–281.14) | 47.32(35.09–76.34) | 136.39(103.44–212.47) | 18.71 | -2.06(-2.49–-1.62) |
| Thailand | 1103.01(803.4–1305.87) | 468.74(345.47–551.71) | 1076.52(755.5–1593.87) | 205.29(143.64–302.08) | -2.4 | -3.47(-3.78–-3.16) |
| Timor-Leste | 8.45(4.7–12.28) | 397.55(232.69–580.22) | 12.38(7.3–18.87) | 278.94(170.96–426.31) | 46.43 | -1.45(-1.76–-1.13) |
| Togo | 70.97(54.8–94.97) | 755.55(593.5–1003.88) | 166.81(115.87–234.83) | 609.53(430.7–842.25) | 135.04 | -0.67(-0.73–-0.61) |
| Tokelau | 0.05(0.03–0.08) | 746.72(501.67–1152.57) | 0.03(0.02–0.05) | 508.59(337.09–755.94) | -31.78 | -1.34(-1.39–-1.28) |
| Tonga | 2.12(1.61–2.7) | 657.29(503.84–827.26) | 2.2(1.57–3.05) | 504.27(363.43–697.2) | 3.54 | -1(-1.15–-0.86) |
| Trinidad and Tobago | 25.19(23.01–28.74) | 534.78(488.6–592.69) | 30.93(22.26–41.89) | 341.27(244.6–463.63) | 22.78 | -1.97(-2.16–-1.77) |
| Tunisia | 30.64(21.68–38.12) | 107.08(77.41–132.47) | 51.17(34.04–71.11) | 75.12(50.45–104.04) | 66.99 | -1.27(-1.31–-1.23) |
| Turkey | 268.31(167.81–339.64) | 126.7(79.93–158.73) | 292.05(202.79–370.12) | 61.84(42.93–78.27) | 8.85 | -2.58(-2.92–-2.24) |
| Turkmenistan | 32.65(29.6–35.91) | 266.86(241.84–291.44) | 62.82(44.16–83.39) | 250.4(176.46–332.23) | 92.43 | 0.52(0.12–0.91) |
| Tuvalu | 0.34(0.22–0.52) | 786.67(499.93–1190.79) | 0.29(0.19–0.44) | 536.14(354.32–816.44) | -15.19 | -1.22(-1.32–-1.12) |
| Uganda | 307.26(208.91–419.71) | 731.46(502.42–987.77) | 796.72(561.36–1070.38) | 756.66(534.93–996.21) | 159.3 | -0.26(-0.53–0.01) |
| Ukraine | 1253.76(853.48–1362.42) | 322.59(221.04–350.79) | 624.07(474.23–856.38) | 171.49(128.53–238.82) | -50.22 | -2.91(-3.16–-2.66) |
| United Arab Emirates | 5.63(3.99–7.81) | 298.16(199.5–420.54) | 28.7(19.98–41.85) | 168.62(117.32–230.98) | 409.36 | -1.77(-2.39–-1.15) |
| United Kingdom | 706.8(617.66–728.87) | 185.8(153.79–191.84) | 433.04(405.96–553.23) | 90.26(85.09–110.18) | -38.73 | -2.43(-2.71–-2.16) |
| United Republic of Tanzania | 601.82(408.1–825.97) | 856.57(584.67–1164.08) | 1179.26(779.28–1695.92) | 702.42(469.38–980.09) | 95.95 | -0.62(-0.76–-0.47) |
| United States of America | 1908.31(1618.24–1997.32) | 126.54(106.2–132.28) | 2247.83(1931.84–2371.26) | 98.77(86.19–104.29) | 17.79 | -0.86(-1.01–-0.7) |
| United States Virgin Islands | 1.92(1.49–2.37) | 358.55(278.49–440.14) | 1.89(1.44–2.42) | 223.88(167.92–293.95) | -1.84 | -1.71(-1.78–-1.65) |
| Uruguay | 63.35(58–68.6) | 355.49(324.79–386.44) | 62.6(55.01–70.46) | 275.05(240.42–311.8) | -1.19 | -1.06(-1.2–-0.91) |
| Uzbekistan | 184.03(167.22–203.19) | 266.46(242.32–292.56) | 433.08(340.38–531.96) | 276.85(219.9–339.05) | 135.34 | -0.13(-0.41–0.14) |
| Vanuatu | 2.16(1.16–3.35) | 539.24(300.64–827.06) | 5.62(2.98–8.8) | 544.48(298.12–842.03) | 160.51 | -0.34(-0.56–-0.13) |
| Venezuela | 410.45(380.94–444.96) | 637.03(591.9–695.22) | 803.73(576.28–1096.19) | 505.7(363.38–688.29) | 95.82 | -1.19(-1.43–-0.95) |
| Viet Nam | 779.09(569.11–1022.64) | 323.87(237.7–426.44) | 1398.83(963.61–1840.65) | 248.94(173.03–325.09) | 79.55 | -0.91(-1.04–-0.79) |
| Yemen | 44.84(20.19–68.79) | 143.74(68.2–214.03) | 104.84(62.42–159.04) | 117.57(72.58–172.6) | 133.82 | -0.79(-0.88–-0.71) |
| Zambia | 235.8(161.9–313.36) | 1181.58(819.63–1553.41) | 431.16(280.43–612.37) | 861.7(568.8–1229.52) | 82.85 | -1.52(-1.73–-1.31) |
| Zimbabwe | 219.31(153.78–282.66) | 859.67(599.63–1106.71) | 481.76(316.22–683.87) | 957.22(639–1349.6) | 119.67 | 1.67(1.09–2.26) |

EAPC: estimated annual percentage change; ASR, age–standardized rate; CI, confidence interval; UI: uncertainty interval.
